# Arithmetic in the signing brain: Differences and similarities in arithmetic processing between deaf signers and hearing non‐signers

**DOI:** 10.1002/jnr.25138

**Published:** 2022-10-19

**Authors:** Josefine Andin, Åsa Elwér, Elina Mäki‐Torkko

**Affiliations:** ^1^ Department of Behavioural Sciences and Learning Linköping University Linköping Sweden; ^2^ Audiological Research Center, Faculty of Medicine and Health Örebro University Örebro Sweden

**Keywords:** arithmetic, deafness, functional magnetic resonance imaging, RRID:SCR_009550, sign language

## Abstract

Deaf signers and hearing non‐signers have previously been shown to recruit partially different brain regions during simple arithmetic. In light of the triple code model, the differences were interpreted as relating to stronger recruitment of the verbal system of numerical processing, that is, left angular and inferior frontal gyrus, in hearing non‐signers, and of the quantity system of numerical processing, that is, right horizontal intraparietal sulcus, for deaf signers. The main aim of the present study was to better understand similarities and differences in the neural correlates supporting arithmetic in deaf compared to hearing individuals. Twenty‐nine adult deaf signers and 29 hearing non‐signers were enrolled in an functional magnetic resonance imaging study of simple and difficult subtraction and multiplication. Brain imaging data were analyzed using whole‐brain analysis, region of interest analysis, and functional connectivity analysis. Although the groups were matched on age, gender, and nonverbal intelligence, the deaf group performed generally poorer than the hearing group in arithmetic. Nevertheless, we found generally similar networks to be involved for both groups, the only exception being the involvement of the left inferior frontal gyrus. This region was activated significantly stronger for the hearing compared to the deaf group but showed stronger functional connectivity with the left superior temporal gyrus in the deaf, compared to the hearing, group. These results lend no support to increased recruitment of the quantity system in deaf signers. Perhaps the reason for performance differences is to be found in other brain regions not included in the original triple code model.

**Significance**

The purpose of this study is to understand similarities and differences in the neural correlates supporting arithmetic in adult deaf signers and hearing non‐signers. We found deaf signers to rely on mainly the same neural networks as hearing non‐signers. However, the involvement of the left inferior frontal gyrus differed between the groups.

## 1 INTRODUCTION

International research has shown that deaf signers lag several years behind hearing peers in mathematics in general (Pagliaro, 2010). However, this is not necessarily the case for all mathematical domains. In a recent study at our lab, Swedish deaf adults were shown to perform on par with hearing peers on simple multiplication and simple subtraction and yet show differences in the recruitment of classical language and magnitude processing brain areas (Andin et al., 2019). Specifically, hearing individuals showed more widespread activation in brain areas that have been related to verbal processing of arithmetic facts in the left inferior frontal gyrus, whereas deaf individuals engaged brain areas that have been related to language‐independent magnitude processing in the right intraparietal sulcus when performing simple arithmetic. This indicates that, compared to hearing non‐signers, deaf signers can successfully make use of processes located to partially different brain areas during simple arithmetic. The main aim of the current study is to further our understanding of similarities and differences in the neural correlates supporting simple and difficult arithmetic in deaf signers compared to hearing non‐signers. As such, this study is a conceptual replication with extension of the work of Andin et al. (2019).

Signed languages are visual, natural, and complete languages in the visuospatial domain (Sandler & Lillo‐Martin, 2006) that support language development much in the same way as spoken languages (Mayberry & Lock, 2003). In 1983, a new curriculum for deaf education was introduced in Sweden and since then, all deaf children and their families are offered sign language courses and support for the child in preschool settings from the age of 1 (LGr 80, 1983). During the 80s, 90s, and 00s, before the introduction of cochlear implants, almost every deaf child in Sweden attended a deaf school during their formal schooling from preschool to high school. This means that they have followed a bilingual curriculum where Swedish sign language has been the main mode of communication and written Swedish is considered a second language (e.g., Bagga‐Gupta, 2004). This has led to favorable linguistic development for Swedish deaf children of both deaf and hearing parents born in the last three decades of the 20th century (Roos, 2006). Therefore, young Swedish adult deaf signers constitute a unique population for whom sign language learning has been optimized (Bagga‐Gupta, 2004). This is in contrast to many other deaf signing populations in countries where oral education of deaf children is still common and where there is a larger variability in preferred language in the deaf population. However, with the contemporary introduction of cochlear implants, the scenery has changed as most children who are born deaf will have access to the auditive domain, and therefore sign language may become used to a lesser extent during childhood. Therefore, it is important to take the opportunity to investigate language modality dependent and independent processes in this deaf young adult population for whom sign language learning has been optimized. This is of both theoretical and practical importance. From a theoretical point of view, this study will broaden knowledge of how cognitive processes are supported by the brain. The present study will also be of importance for future studies on other populations since it generates knowledge about cognitive functions in individuals that are solely dependent on the visual modality for communication. Such knowledge will be important when formulating hypotheses about cognitive functions in individuals that rely on both the visual and the auditory modality, especially individuals with cochlear implants. Because the use of imaging techniques in the cochlear implanted population is limited, it is important to explore the signing brain using, for example, functional magnetic resonance imaging (fMRI) to generate testable hypotheses that can be explored with less elaborate imaging techniques such as functional near‐infrared spectroscopy (fNIRS), which is compatible with cochlear implants. Furthermore, there will always be individuals for whom cochlear implants are not an option, for example, due to missing cochleae, medical or financial reasons. Therefore, knowledge generated by this study will be valuable in informing teaching strategies for this small group. Furthermore, the present study could also add valuable information in relation to other groups with mathematical difficulties, such as developmental as well as acquired dyscalculia, where it might be difficult to disentangle linguistic and mathematical aspects.

Arithmetic concerns the basic operations of numbers, that is, addition, subtraction, multiplication, and division. Evidence suggests that both verbal and quantity competencies are involved when engaging with arithmetic operations (Dehaene et al., 2003; Lee & Kang, 2002; Zhou et al., 2018). Quantity competencies involve magnitude manipulations along a mental analog number line and verbal competencies come into play when pre‐learned facts are retrieved from long term memory. These two competencies are engaged to different degrees depending on the operation at hand. In this sense, the operations can be considered to represent a continuum with multiplication and subtraction representing the extremes, while addition and division are placed in between the two. Multiplication primarily taxes verbal competence as multiplication tables typically are learned by rote learning and can be retrieved by arithmetic fact retrieval. Subtraction, on the other hand, mainly taxes quantity competencies as subtraction is less likely to be learned by rote learning and instead requires manipulation along the mental number line. The exception being simple subtraction tasks, where retrieval strategies may also be used (e.g., Andin et al., 2019; Simon et al., 2002). Several studies have found that verbal and quantity competencies are supported by partially different neural correlates (e.g., Dehaene et al., 2003; Prado et al., 2011). The rationale for including multiplication and subtraction in most neuroimaging studies (including the present) is to target these two competencies.

Perhaps the most influential model of number processing, the triple‐code model, proposes three different systems for number processing of which the verbal and quantity system are two, the third being the visuospatial attentional system (Dehaene et al., 2003). Although recent brain imaging research has challenged the validity of the model, the verbal and the quantity systems are consistently implicated in arithmetic processing (for review see Arsalidou et al., 2018; Arsalidou & Taylor, 2011).

The *verbal system* includes verbal representations of numbers and is especially involved in arithmetic fact retrieval. Deaf individuals have been found to perform worse than hearing individuals in a number of tasks related to the verbal system, for example, relational statements (Kelly et al., 2003; Serrano Pau, 1995), arithmetic word problems that require reading (Hyde et al., 2003), fractions (Titus, 1995) and multiplication (Andin et al., 2014; Nunes et al., 2009). According to the original triple‐code model, the left angular gyrus was suggested to be the main region for the verbal system (Dehaene et al., 2003), which is further supported by several other studies (Grabner et al., 2009, 2011; Price & Ansari, 2011). In the left angular gyrus, activation has been found for tasks of exact compared to approximate calculation, small compared to large numbers, and multiplication compared to subtraction (for a review see Dehaene et al., 2003). Activation has also been reported to decrease with increased complexity of the task at hand (Artemenko et al., 2019) and to increase with mathematical competence (Grabner et al., 2007). However, several recent studies have failed to find activation for arithmetic tasks in this region (Grabner et al., 2013; Wu et al., 2009). In a recent study, when using the whole left angular gyrus as a region of interest it was deactivated for both hearing and deaf individuals for simple subtraction and multiplication compared to a baseline task (Andin et al., 2019). However, the posterior and the anterior portion of left angular gyrus show distinct differences in terms of both structural and functional connectivity (Uddin et al., 2010). The posterior part of the left angular gyrus constitutes the lateral parietal node of the default mode network, meaning that this region is activated during rest and deactivated during tasks that require attention to shift from default mode to task. Accordingly, the engagement of the posterior left angular gyrus during arithmetic processing might be related to difficulty modulations rather than to arithmetic tasks per se (Wu et al., 2009). The anterior left angular gyrus, on the other hand, does not seem to be as strongly connected to the default mode network and shows less or no deactivation during arithmetic tasks. Uddin et al. (2010) argue that dividing the left angular gyrus into two different regions is important for understanding how these regions are engaged during numerical cognition. Despite inconsistency between studies, it is uncontested that left angular gyrus is of importance for arithmetic processing. However, another line of research shows that, although the left angular gyrus is important during arithmetic fact retrieval, its role is merely to mediate and allocate attention during retrieval (Bloechle et al., 2016; Klein et al., 2019). Instead, there are several studies showing that the region responsible for the encoding and retrieval of arithmetic facts is the hippocampus (Bloechle et al., 2016; Klein et al., 2016). This notion is supported by analyses of functional connectivity between the hippocampus and the left angular gyrus, where Klein et al. (2016) has shown that the angular gyrus and the hippocampus are functionally connected for fact retrieval tasks, but not for magnitude processing in a hearing population. If the role of the angular gyrus is to mediate hippocampal function it is likely that this connection is less apparent in deaf individuals. However, as the hippocampus is a general hub for memory formation (e.g., Eichenbaum, 2004) and deaf individuals do not have any specific memory deficit, it is possible that the hippocampus may be connected to other regions, such as the right intraparietal sulcus, which would indicate a different route to fact retrieval via magnitude processing in deaf signers.

A further extension of the verbal system, from the left angular gyrus towards larger parts of the left‐lateralized perisylvian network has been suggested by several researchers (Arsalidou & Taylor, 2011; Fedorenko et al., 2012; Skagenholt et al., 2018). In particular, the left inferior frontal gyrus, which is generally activated during verbal tasks, seems like a promising region for arithmetic reasoning. This region has been found to be activated during size comparison task for both digits and number words compared to letters (Skagenholt et al., 2018), difficult versus simple arithmetic tasks (Fedorenko et al., 2012), two‐digit versus one‐digit multiplication problems (Soltanlou et al., 2017) and carrying and borrowing in high versus low performing individuals (Artemenko et al., 2018). Recent studies have further shown that resting‐state functional connectivity between the left inferior frontal gyrus and the angular gyrus is stronger for individuals that are proficient in arithmetic (Skagerlund et al., 2019). As deaf individuals seem to show less activation in the verbal system during arithmetic, we expect this connection to be less apparent in deaf compared to hearing individuals.

The *quantity system* is primarily involved in magnitude manipulation along the analog mental number line. For tasks associated with this system, there is no evidence of poorer performance for deaf individuals: Deaf and hearing children perform at similar levels in basic competencies such as subitizing (Bull et al., 2006), magnitude processing (Bull et al., 2006), and number comparisons (Bull et al., 2005) and deaf adults perform on par with hearing adults in subtraction (Andin et al., 2014). The quantity system was originally located to the bilateral horizontal portion of the intraparietal sulcus (Dehaene et al., 2003). This region has been shown to be activated more for subtraction than multiplication, more for approximate than exact calculation and more for number words compared to other types of words. Thus, it has been suggested that this region is a primary neural correlate for magnitude processing (Skagenholt et al., 2018; Sokolowski et al., 2017). Connectivity studies have suggested that connectivity between the right intraparietal sulcus and several different brain regions is modulated by arithmetic competence (Jolles et al., 2016; Rosenberg‐Lee et al., 2015; Skagerlund et al., 2019). Skagerlund et al. (2019) showed that individuals with high arithmetic proficiency had stronger connectivity between the right intraparietal sulcus and the left frontal regions as well as the left supramarginal gyrus, whereas connection to right frontal regions was associated with poorer arithmetic competence. For children with developmental dyscalculia, Jolles et al. (2016) and Rosenberg‐Lee et al. (2015) found the right intraparietal sulcus to be hyperconnected to several other regions, including the bilateral angular gyrus, leading to the suggestion that these children have fundamental differences in their parietal organization compared to typically developing children. Although both deaf individuals and individuals with developmental dyscalculia perform under par in arithmetic we do not know if the basis for the problems share mechanism in the two groups. In the present study, we will investigate how connectivity from this region differs between deaf and hearing individuals.

The neuronal correlates underlying number processing in deaf individuals are poorly understood. To the best of our knowledge, there are only three imaging studies on number processing in deaf signers (Andin et al., 2018, 2019; Masataka et al., 2006). Masataka et al. (2006) showed that neural systems similar to those found for calculation in hearing individuals are activated when deaf individuals learned the signed numerals of another sign language than their own. In a number and letter order task in Andin et al. (2018), there was a tendency towards stronger activation of the right horizontal intraparietal sulcus in deaf compared to hearing individuals. In the only study so far investigating neural correlates of arithmetic processing in deaf signers, the results showed that the right horizontal intraparietal sulcus was significantly recruited for both deaf and hearing individuals during subtraction, but only for deaf individuals during simple multiplication (Andin et al., 2019). The activation differed significantly between groups for simple multiplication, whereas there were no group differences for simple subtraction. We also showed that while both deaf and hearing individuals show significant activation for multiplication in the left inferior frontal gyrus, only hearing individuals show significant activation for subtraction (between‐group analyses were not significant in either comparison). Importantly, there was no significant difference in performance in either response time or accuracy for subtraction or multiplication. As mentioned above, the left angular gyrus was not found to be significantly activated during simple arithmetic for either group (Andin et al., 2019). However, it should be noted that the stimulus material was designed to be used for different tasks including digit and letter order (Andin et al., 2018) and phonology (Andin et al., 2019). Therefore, the stimulus material required the use of working memory and other cognitive competencies. In the present study, will we use stimuli consisting of pure arithmetic tasks to be able to investigate arithmetic processes in a more direct way.

In domains other than arithmetic, it has been shown that deaf individuals have stronger connectivity to and from the superior and middle temporal cortices for task versus no task (Malaia et al., 2014), during reading (Hirshorn et al., 2014) and during resting state (Cardin et al., 2018). Whether connectivity between regions involved in arithmetic and temporal cortex differs between deaf and hearing individuals is unknown. Further, as temporal regions are found to be involved in linguistic and cognitive tasks to a higher degree in deaf compared to hearing individuals (Cardin et al., 2018; Twomey et al., 2017), it is possible that temporal regions have a role in arithmetic processing in deaf individuals, which would show up as connections between the right intraparietal sulcus and temporal regions as well as activation of the temporal regions in the whole‐brain analysis.

**FIGURE 1:**
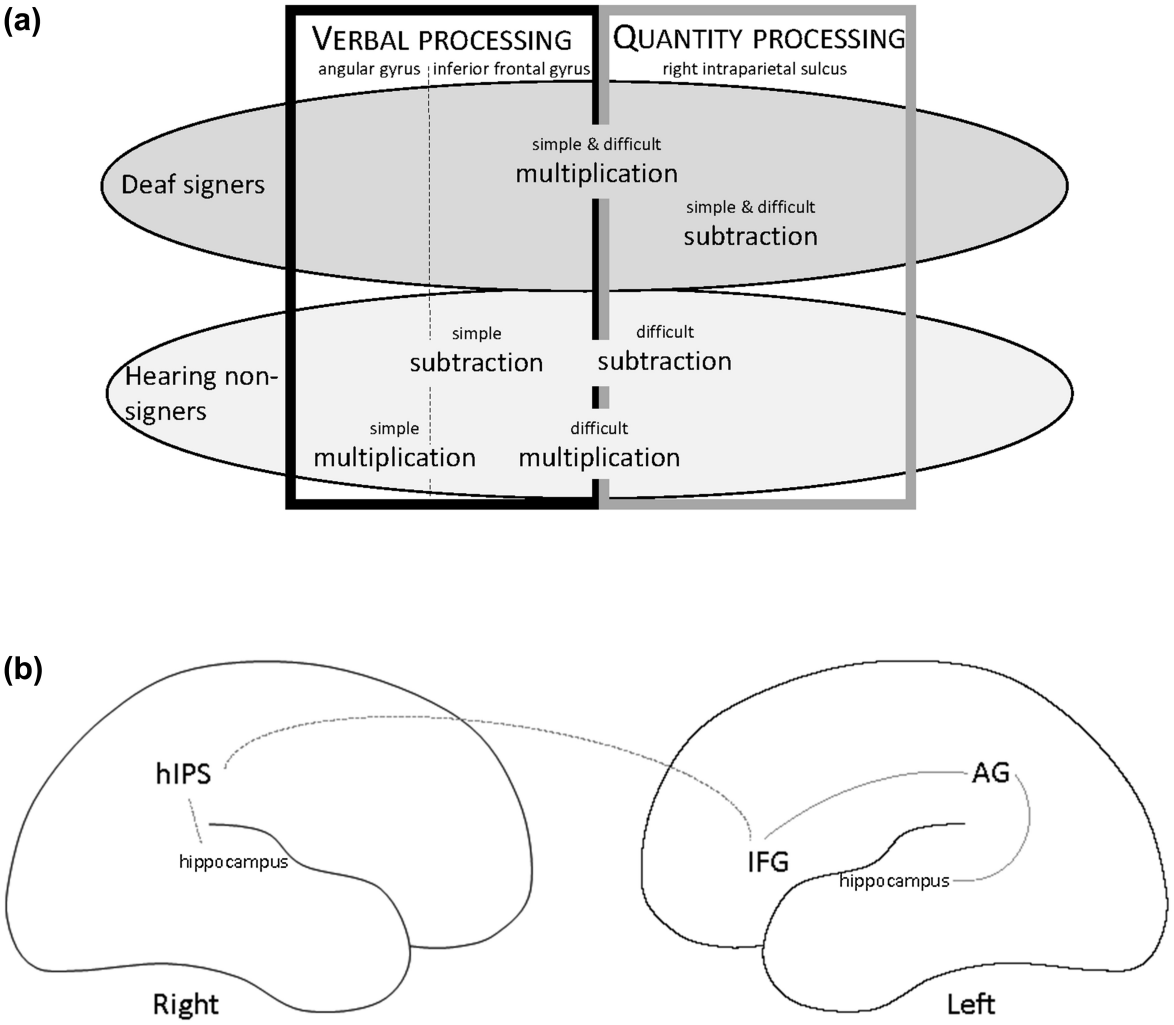
Overview of predictions tested in the present study. (a) Predictions primarily derived from Andin et al. (2019) suggest that deaf signers recruit regions related to quantity processing for simple subtraction (activation in the right horizontal intraparietal sulcus but not in the left inferior frontal gyrus and left angular gyrus/hippocampus) and regions related to both verbal and quantity processing during simple multiplication (activation in both the right horizontal intraparietal sulcus, left inferior frontal gyrus but probably not in the left angular gyrus/hippocampus), whereas hearing non‐signers recruit regions related to verbal processing during simple multiplication (activation of the left inferior frontal gyrus and left angular gyrus/hippocampus but not the right horizontal intraparietal sulcus) and regions related to both verbal and quantity processing duringsimple subtraction (activation of both the left inferior frontal gyrus, left angular gyrus/hippocampus and right horizontal intraparietal sulcus). Difficult multiplication will recruit both systems in both groups, whereas difficlt subtraction will recruit mainly quantity processing in both groups. (b) Task‐based connectivity analyses will show connectivity between the angular gyrus, inferior frontal gyrus, and hippocampus in hearing individuals (gray lines), whereas in deaf individuals there will be connections between the right intraparietal sulcus and the inferior frontal gyrus and possibly the hippocampus (dotted gray line). AG, angular gyrus; hIPS, horizontal portion of the intraparietal sulcus; IFG, inferior frontal gyrus.

Combining results from behavioral and imaging studies suggests that, when engaging in arithmetic tasks, deaf signers successfully make use of different strategies and neuronal regions compared to hearing non‐signers. These results call for further exploration of the hypotheses that deaf signers recruit regions related to quantity processing for simple subtraction and regions related to verbal and quantity processing during simple multiplication, whereas hearing non‐signers recruit regions related to verbal processing during simple multiplication and regions related to both verbal and quantity processing during simple subtraction. There are no studies on the neural correlates of difficult arithmetic in deaf populations. However, for hearing individuals it is expected that they will recruit more of the quantitative system for multiplication beyond the rote‐learned multiplication tables, which are not retrieved by arithmetic fact retrieval, but possibly through approximation strategies (Dehaene, 1992; Dehaene et al., 2003; Ganor‐Stern, 2016; Soltanlou et al., 2017). Further, several studies have shown that the triple code model is a reliable model of numerical cognition, although with some changes, for hearing individuals. In this study, we will investigate the universality of the model in the deaf population that uses a different language modality, that is, visuospatial rather than auditory language. See Figure 1 for an overview of these hypotheses.

### 1.1 Aim

This study is a conceptual replication and extension of Andin et al. (2019) with more power. The conceptual replication includes simple multiplication and subtraction, but with a stimulus material more suited for analyses of arithmetic processes and the extension includes difficult multiplication and subtraction as well as connectivity analyses. The overarching aim of the present study is to understand similarities and differences in neural correlates supporting arithmetic in adult deaf signers and hearing non‐signers. The primary objective is to investigate the role of the right horizontal intraparietal gyrus, the left inferior frontal gyrus, the left angular gyrus, and the hippocampus during simple and difficult arithmetic. In detail, we predict the following:

1. Deaf signers will show activation in:
  - the right horizontal intraparietal sulcus for both simple and difficult subtraction and multiplication,
  - the left inferior frontal gyrus and left angular gyrus for multiplication, and
  - the hippocampus for simple multiplication and subtraction.
2. Hearing non‐signers will show activation in:
  - the right horizontal intraparietal sulcus for difficult subtraction and difficult multiplication,
  - the left inferior frontal gyrus and left angular gyrus for multiplication and subtraction, and
  - the hippocampus for simple multiplication and subtraction.
3. There will be differences between groups such that:
  - deaf signers will show stronger activation compared to hearing non‐signers in the right horizontal intraparietal sulcus for simple multiplication,
  - hearing non‐signers will show stronger activation compared to deaf signers in the left inferior frontal gyrus and possibly also in the left angular gyrus for subtraction and simple multiplication, and
  - hearing non‐signers will show stronger activation compared to deaf signers in the hippocampus for multiplication.
4. There will be group differences in how the involved regions are connected. We expect to find functional connectivity between:
  - the right intraparietal sulcus, the left inferior frontal gyrus and possibly the hippocampus for the deaf group, and
  - the left angular gyrus, the left inferior frontal gyrus and, the hippocampus in hearing individuals, whereas such connectivity will be weaker or not found at all for deaf individuals.

The second objective is to explore which other brain regions support arithmetic in deaf signers. We expect to find:

5 mainly similar networks recruited for both groups at whole‐brain analyses,
6 the superior temporal regions (primary and secondary auditory cortex) to be involved in arithmetic for deaf individuals, and
7 stronger connectivity from the arithmetic network to the superior temporal regions for deaf signers compared to hearing non‐signers.

## 2 METHODS

### 2.1 Power analysis

To the best of our knowledge, there are two main ways of performing power analyses in fMRI studies. The first is to do a pilot study of the design on a small sample of participants and the second is to use statistical maps from similar studies. In this project, pilot work will be very difficult because the population of deaf signers in Sweden is so small that it will be impossible to find enough participants for both a pilot and the main study. It would be possible to do a pilot study on hearing individuals, but that would not be helpful as we are interested in group differences. The other option is to use statistical maps from a similar study. The only previous fMRI‐study on arithmetic in deaf signers is Andin et al. (2019) and in that study, we did not investigate difficult arithmetic tasks. However, as this is the only study available, fMRI power analyses were performed using that study. The fMRIpower software (fmripower.org, Mumford & Nichols, 2008) was used to estimate the power needed to detect significant activation within specific regions of interest with the effect size given in SD‐units (equivalent to the Cohen's *d* measure). *T*‐maps were used for the power estimations, as this tool does not allow for power calculations of more complex designs. We performed power calculations for each group individually (prediction 1 and 2) using one‐sample *t*‐tests (alpha level 0.05) and for group comparisons (prediction 3) using two‐sample *t*‐tests (alpha level of 0.05).

Related to prediction 1, we will, for the deaf group, have at least 80% power to

- detect an effect size of 0.83 SD‐units with 11 participants for multiplication in the right intraparietal sulcus,
- detect an effect size of 0.67 SD‐units with 17 participants for subtraction in the right intraparietal sulcus, and
- detect and effect size of 0.35 SD‐units with 16 participants for multiplication in the left inferior frontal sulcus.

Related to prediction 2, we will, for the hearing group, have at least 80% power to

- detect an effect size of 0.48 SD‐units with 27 participants for multiplication in the left inferior frontal gyrus and
- detect an effect size of 0.47 SD‐units with 28 participants for subtraction in the left inferior frontal gyrus.

Related to prediction 3, we will, for group comparisons, have at least 80% power to

- detect an effect size of 0.82 with 18 participants per group in the right intraparietal sulcus for multiplication and
- detect an effect size of 0.61 with 34 participants per group in the left inferior frontal gyrus for multiplication.^1^

For the other contrasts, it was not possible to calculate power due to deactivation (in the left angular gyrus and partly the hippocampus) and in some cases, the sample size needed was too high to be reasonable for this study. Concerning the hippocampus, the study, on which the power calculations are based on, did not investigate direct fact retrieval, and as fact retrieval is the proposed action of the hippocampus, it is still possible that we would be able to find effects there. Further, based on these calculations, we did not have power to detect effects relating to subtraction in any region for hearing individuals. This can be a problem, but it should be noted that these calculations are only performed for simple multiplication and subtraction. We expected effects of difficult subtraction to result in stronger activations in the right intraparietal sulcus. Based on these estimates and the fact that the main region of interest is the horizontal intraparietal sulcus, we aimed to scan 34 participants per group. Excluded participants were replaced.

However, it should be noted that the current research design varies considerably from the design used in the power estimations. The present study is better optimized with fewer conditions (the Andin et al., 2019—study also included other conditions than arithmetic) and more repetitions of each condition. The magnetic field of the MR‐scanner in the present study will be higher (3 T compared to 1.5 T), which will improve the signal‐to‐noise ratio and thus lead to higher power. Further, as deaf signers belong to a population that is difficult to recruit and the design in the present study is better optimized to find group differences, we believe that fewer participants will suffice. Based on this and on economical constraints, we planned to finalize the data collection by the end of December 2020 even if the aim of 34 participants per group has not been reached. However, due to the covid‐19 pandemic we extended the data collection to September 2021 and landed on 29 participants per group. However, it should be noted that with a group size of 29, we have the power to detect all but the effect of multiplication in the left inferior frontal gyrus. Further, small groups are commonly used in fMRI‐studies on deaf signers even when they belong to larger sign language populations, such British and American sign language users.

### 2.2 Participants

Twenty‐nine deaf early signers and twenty‐nine hearing non‐signers were included in the study. Thirty deaf early signers were recruited to participate in the study (age *M* = 35.6, SD = 6.1, range = 25–46). One participant was not able to see the screen and was removed from all further analyses; hence, the group consists of twenty‐nine individuals. Inclusion criteria for the deaf participants were prelingual deafness, using Swedish sign language as the main mode of communication from early childhood (before age of 3), having been enrolled in bilingual schooling and not having a cochlear implant. All participants reported native or native‐like sign language skills. One participant reported using another sign language next to Swedish and one participant reported using spoken and sign language, the rest reported mainly using sign language. All deaf participants attended deaf school at least during compulsory schooling (9–10 years). The age range of the participants was 25 to 46 years of age (born 1976 or later), allowing us to take advantage of the unique sign language experience of deaf adults that were enrolled in school after the introduction of the bilingual curriculum in 1983 (see Table 1 for a summary of inclusion and exclusion criteria). The focus of the study was to investigate arithmetic processing in signing individuals. However, it cannot be precluded that different types of hearing deficits/deafness affect neurobiological networks. Therefore, we asked the participants for the cause of their deafness; however, most participants did not know, and therefore we did not analyze this further.

**TABLE 1:** Summary of inclusion and exclusion criteria for participation

	Deaf signers	Common to both groups	Hearing non‐signers
Inclusion criteria			
Primary language	Swedish sign language		Spoken Swedish
Schooling	Bilingual		Mainstream
Hearing status	Deaf		Hearing
Age of acquisition for primary language		<3 years of age	
Handedness		Right‐handed	
Year of birth		1975–2002^a^	
Exclusion criteria			
Hearing aids	Cochlear implants and hearing aids^b^		Cochlear implants and hearing aids
Nonverbal intelligence		<2 SD from norm group mean	
Other conditions		Neurological or psychiatric conditions	
Scanning		Movement more than 3 mm movement in *x*, *y*, *z* or 3° in pitch, yaw, roll	

^a^ Participants had to be at least 18 years old, that is, those tested during 2019 were born before 2001 and those tested during 2020 were born before 2002.

^b^ If used to access spoken language. Participants using hearing aids for alarm purposes were included.

Twenty‐nine hearing non‐signers matched to the deaf early signer group for age (*M* = 33.3, SD = 9.7, range = 18–46), education level, nonverbal intelligence and gender were recruited. These participants were unfamiliar with Swedish sign language and had Swedish as their first language. Nonverbal intelligence was tested using the visual puzzle subtest from the Wechsler adult intelligence scale (Fourth version, Wechsler, 2008), which has been shown to be highly correlated with general intelligence. No participants in either group performed more than two standard deviations below the mean of the norm group. There were no differences between groups in age, *t*(47.1) = 1.07, *p* = .288 (degrees of freedom was adjusted due to unequal variance), nonverbal intelligence, *t*(56) = 0.92, *p* = .360, gender, *χ* ^2^ = 0.069, *p* = .792 or level of education, *χ* ^2^ = 1.94, *p* = .379. Descriptive data are presented in Table 2.

**TABLE 2:** Group demographics and general cognitive ability

	Deaf signers		Hearing non‐signers		*t*	*p*
	*M*	SD	*M*	SD		
Age	35.6	6.1	33.3	9.7	1.07	.288
Visual puzzles	17.4	4.4	18.5	4.2	0.98	.333
	*n*		*n*		*χ* ^*2*^	*p*
Gender						
Female	16		15		0.069	.792
Male	13		14			
Highest level of education						
Elementary	0		1		1.94	.379
High school	14		10	
University	15		16	

All participants were right‐handed with no history of neurological or psychological conditions. Instructions were given orally for hearing participants and in Swedish sign language for deaf participants (through a signing research assistant). Written informed consent was signed by all participants. Information about the project and the rights of the participants were given in Swedish, Swedish sign language and in written form before the consent form was signed. The project was approved by the Swedish Ethical Review Authority (Dnr: 2019‐00896). The participants were paid SEK 1000 for their participation (app. $ 100).

### 2.3 Procedure

Upon inclusion in the project, participants filled in an online form including questions about their education, work situation, age and age of acquisition of Swedish sign language. The participants that fulfilled the inclusion criteria were invited to the Stockholm University Brain Imaging Centre (SUBIC), where they performed fMRI testing, tests of arithmetic, working memory and nonverbal intelligence.

### 2.4 Brain imaging and analyses

#### 2.4.1 Stimulus material

The material consisted of four experimental and one baseline condition: simple multiplication (operands <10, e.g., 03 × 05 = 15), difficult multiplication (one operand >10, e.g., 22 × 03 = 66), simple subtraction (all numbers <10, e.g., 08–06 = 02), difficult subtraction (answers >10, the second operand <10 and with borrowing procedure, e.g., 41–08 = 33) and baseline (same digits, e.g., 03 = 03 = 03). All operands and answers were < 100. The task was to identify, by button press, if the stated equation was correct or not (correct in the baseline task refers to the same digit at all places, for example, 03 = 03 = 03 is correct, but 03 = 07 = 03 is incorrect). The distance between incorrect and correct answers was balanced over conditions and varied between −3 to +3 from the correct answer in all conditions.

There were 64 unique trials in each condition. In all conditions, the proportion of targets and foils were 1:1 (32 with correct and 32 with incorrect answers). Blocks with performance below 50% were excluded from further analyses. Using 0 before single digits equalized the visual appearance of the stimulus, leading to comparable activation of visual brain regions. Behavioral pilot testing of the material has shown that although the participants experienced the 0:s before the number (e.g., 03) as distracting, it did not interfere with accuracy or response time.

The tasks of interest were the arithmetic tasks. The baseline condition was only used, during fMRI analysis, to subtract processes common for all tasks, that is, visual input as well as button‐press, such that the fMRI analysis was focused on task‐specific activation. (Sometimes it turns out that the baseline task is not as optimal as has been anticipated. If we had experienced problems with the baseline task, we would have been able to contrast the other tasks to rest instead. However, the baseline task turned out to work well.)

The participants practiced the tasks off‐line before entering the scanner, with materials not used in the scanner. In addition to the fMRI data, behavioral data from the button pressing were collected and analyzed. Stimuli were presented using the Psychtoolbox running under MatLab 2019a (The MathWorks Inc., Natick, MA).

#### 2.4.2 Experimental design

The experiment was set up as a 2 × 2 × 2 factorial blocked design with group (deaf, hearing) as a between‐subjects factor and equation type and difficulty as within‐subject factors, where ‘equation type’ refers to subtraction or multiplication and ‘difficulty’ to simple or difficult operations. The blocked design included two runs with a total of sixty‐four trials in each of the five conditions. There were eight trials in each block and four blocks of each equation type per run. During each trial, the stimulus was displayed for 3000 ms with 50 ms intertrial‐pause, such that each block lasted for 24,350 ms. To guard against differences in visual stimulation, the stimuli were visible during the 3000 ms regardless of when the answer was given. Button press with the right thumb was used.

**FIGURE 2:**
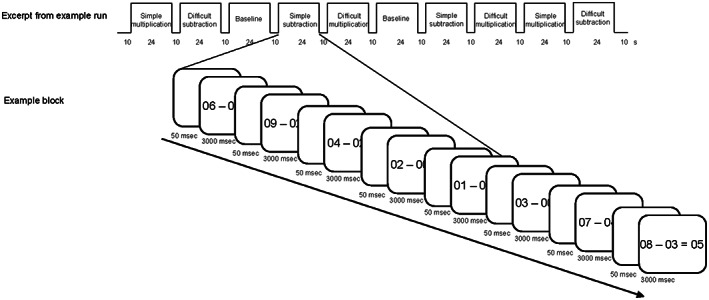
Schematic representation of an excerpt from a scanning run and an example of stimulus display and timing within a block of simple subtraction.

The between‐block interval was 10,000 ms. Each run started with a blank screen for approximately 10 s to allow for magnetization to stabilize to steady‐state and ended with a blank screen for approximately 10 s to capture the hemodynamic response of the last block. Hence, the experiment consisted of two approximately 12 min long runs. An overview of the design is shown in Figure 2. Within each task type, the 64 different trials were pseudo‐randomized into eight blocks, in which the proportion of correct to incorrect trials was between 3:5 to 5:3 (i.e., 3, 4 or 5 correct trials in each block, to prevent expectation effects). The blocks were presented pseudo‐randomly and permuted within each run, such that every block type was equally likely to appear as starting block in a run and such that every block type was presented once within each epoch (i.e., all block types appeared once before any block type was repeated within each run). There were as many unique compositions of blocks during the two runs as there were participants in one group, that is, the same block order was used for the first participant in both groups, a new order was used for the second participant in both groups, and so on. To further avoid order effects, there was always a block order for one participant (of each group) that was the reverse of the block order of another participant (i.e., participant no 2 in each group was given the reverse block order compared to participant no 1 in each group, participant no 4 in each group was given the reverse of participant no 3 in each group). In total, all participants performed the same 320 trials (64 trials for each of the five task types) but in different orders.

After the two runs of arithmetic, included in this study, the participants performed two runs of geometry tasks. The geometry part will form a separate exploratory publication.

#### 2.4.3 Data acquisition

MRI data were acquired on a 3.0 T scanner (Siemens Magnetom Prisma, Siemens Healthcare GmbH), equipped with a 64‐channel head coil, at Stockholm University Brain Imaging Center (Stockholm, Sweden: SUBIC). Functional data were collected, during continuous scanning, using a BOLD EPI sequence (TE/TR = 30/1760 ms, FA = 70°, oblique axial slice orientation, FOV = 192 × 192 mm, slice thickness = 2, in‐plane resolution = 2 × 2 mm, number of slices = 58, GRAPPA acc. = 2, SMS acc. = 2). We also collected T1‐weighted structural image (3D MPRAGE, TI/TE/TR = 900/2.98/2300 ms, FA = 9°, oblique axial slice orientation, FOV = 256 × 256 mm, 256 × 256 × 208 acquisition matrix, 1 × 1 × 1 mm^3^ voxels, GRAPPA acc. factor = 2, scan time = 5:21) that were used for normalizing the functional data to MNI space.

There were no incidental findings during scanning or image analyses. In the case of incidental findings during scanning, these would have been referred to the radiologist engaged by Stockholm Brain University Imaging Centre. The radiologist would have examined the findings and if necessary, referred the participant to a primary care physician for further medical examination. Before scanning, participants were informed about what incidental finding is and how such findings would be handled.

#### 2.4.4 Statistical analyses of imaging data

Preprocessing and analysis was performed using statistical parametric mapping packages (SPM12; Wellcome Trust Centre for Neuroimaging, London, UK) and CONN toolbox (version 20b; www.nitrc.org/projects/conn, Whitfield‐Gabrieli & Nieto‐Castanon, 2012) running under MatLab R2018a (The MathWorks Inc., Natick, MA).

##### 2.4.4.1 Preprocessing

Before SPM‐preprocessing, the Dicom‐images from the MR‐scanner was converted to NIFTI format using the Dicom‐to‐nifti converter in MRIcron (Rorden & Brett, 2000). Preprocessing was performed following standard SPM12 procedure and include (1) realignment, which is performed to correct for subject motion between volumes, (2) coregistration of the structural and functional images, (3) segmentation and spatial normalization to match the geometry of subject brain to standard space, (4) normalization of functional and structural images where a segmented deformation field from the previous step is applied to all functional and structural images, and (5) spatial smoothing to increase sensitivity using an 8 mm FWHM Gaussian kernel. Individuals with more than 3 mm movement in *x*, *y*, *z* or 3° in pitch, yaw, roll, were excluded or partially excluded (i.e., if movement was restricted to one run, the other run was included in the analyses and if movement was restricted to a smaller part of a run, the rest of the run was in some cases kept). In total, after removing blocks with performance below 50% and runs/blocks with excessive moment, an average of 33 blocks per participant (out of 40) were kept. There was no significant difference in number of removed blocks between groups, *F*(1,56) = 0.68, *p* = .413, $\eta_{p}^{2}$ = 0.012.

To investigate the primary objective of this study (prediction 1–4) region of interest (ROI) and connectivity analyses were performed. The secondary exploratory objective was tested using whole‐brain analyses (prediction 5–6) and functional connectivity analyses (prediction 7).

##### 2.4.4.2 Whole‐brain analyses

Whole‐brain analyses were conducted by fitting a general linear model with regressors representing the five different task types as well as the six motion parameters derived from the realignment procedure. At first level, individual statistical parametric map images pertaining to each of the four experimental tasks were contrasted with the baseline task. These images were analyzed, individually for each participant, through a 2 × 2 [equation type × difficulty] ANOVA. From the ANOVA, contrast files pertaining to the main effect of equation type and difficulty as well as the interaction effect between equation type and difficulty were brought into second‐level analyses where one‐sample *t*‐tests were performed separately for deaf signers and hearing non‐signers. Finally, second‐level between‐group analyses were performed by bringing the individual contrast files into one‐sample *t*‐tests for the main effect of equation type and difficulty and in two‐sample *t*‐tests for the main effect of group and interaction effects. To investigate the nature of significant interaction effects, simple main effect analyses were performed using the interaction activation as an inclusive mask.

As we expected similar activation in both groups, at least for difficult multiplication (see Figure 1a), conjunction analyses in equation type and difficulty across groups were performed to answer questions about similarities in activation between groups. Significant conjunction indicates that the contrasts evaluated are consistently high and jointly significant across the tested conditions (Friston et al., 2005).

Activation was considered as significant if *p*_fwe_ <.05 at peak level. As brain activation data from deaf individuals are known to be very heterogeneous, it can be difficult to obtain activation that survives the conservative family‐wise error correction at peak level. Therefore, cluster‐level analyses at *p*_fwe_ <.05 are also reported.

##### 2.4.4.3 Region of interest analyses

To investigate the specific hypotheses, ROI‐analyses were performed. Regions of interest were the anterior and posterior portion of the left angular gyrus, the whole angular gyrus, the right horizontal intraparietal sulcus, the left inferior frontal gyrus, and the bilateral hippocampus as defined by the SPM Anatomy Toolbox (version 2.2b; Eickhoff et al., 2005). Since the literature on the angular gyrus is diverging, we analyzed both anterior, posterior, and the whole angular gyrus. ROI mean values were obtained for each contrast (task minus baseline, as described above under “whole‐brain analyses”) from each ROI for every individual, again using the SPM Anatomy Toolbox. Mean values were then entered into a 2 × 2 × 2 (equation type × difficulty × group) ANOVA in SPSS statistics 26 (IBM, SPSS Statistics, version 26, IBM Corporation, New York, USA). Confidence intervals, obtained from the ANOVA, were used to answer predictions 1 and 2, that is, whether there was significant activation (mean ROI values ≠ 0) separately for the two groups.

##### 2.4.4.4 Connectivity analyses

Task‐based functional connectivity was analyzed using Conn—functional connectivity toolbox (version 20b; www.nitrc.org/projects/conn, RRID:SCR_009550). Correlational analyses between the BOLD signal in the seed ROIs and all ROIs and networks included in Conn were performed to obtain ROI‐to‐ROI connectivity estimations for each participant. The seed ROIs were the same as in the ROI‐analyses described above, that is, ROIs from the anatomy toolbox were entered as seed‐ROIs in Conn. This choice is because the ROIs available in Conn do not include the subdivision of the angular gyrus. First‐level covariates included realignment parameters. Second‐level covariates were group (deaf/hearing), age, and raw score from the nonverbal intelligence test. Denoising, including using standard Conn recommendations, was carried out to remove unwanted motion and artifacts from the BOLD signal before connectivity measures were computed. High‐pass filtering at 0.008 Hz was also applied. At second‐level, within‐group connectivity, as well as between‐group connectivity, was analyzed for the four tasks.

### 2.5 Behavioral tests and analyses

Behavioral measures of working memory, arithmetic skill, and in‐scanner performance were collected and used to ensure that potential group differences in brain activation were not due to differences in these skills. All behavioral data were analyzed using SPSS statistics 26 (IBM, SPSS Statistics, version 26, IBM Corporation, New York, USA).

#### 2.5.1 Working memory

To assess the participant's working memory ability, we conducted a computerized version of the Corsi block‐tapping test (Corsi, 1973). In this test, nine colored squares appear on the screen. A sequence of blocks changes color from white to green for 500 ms (inter‐stimulus interval 500 ms). After the last block in the sequence lights up, a signal is given that informs the participant to start clicking at the squares in the same order, using a computer mouse. In the first sequence, two squares are included in the sequence. If the participant correctly reproduces the sequence, the next sequence is increased by one. If the participant fails to reproduce the sequence, they are given a second attempt at the same sequence length. The test ends after two attempts in which the participant fails to reproduce a sequence. The span length was defined by the number of squares included in the last sequence the participant reproduces correctly. The test was implemented in Psychtoolbox running under MatLab 2019a (The MathWorks Inc., Natick, MA).

#### 2.5.2 Arithmetic skills

To control for differences in performance between groups, arithmetic skills were tested using a computerized version of the Skagerlund arithmetic test (Skagerlund et al., 2019). The test includes four subtests, one for each equation type (addition, subtraction, multiplication, and division). In each subtest, the participant is asked to complete as many arithmetic problems as possible within 120 s. The difficulty of the problems increase within each subtest by increasing the number of digits included or by requiring borrowing and carrying. Each subtest includes 54 problems, except for the division subtest that contains 27 problems. The total score is the number of correctly completed problems, with a maximum score of 189. The test was implemented in Psychtoolbox running under MatLab 2019a (The MathWorks Inc., Natick, MA). Results were analyzed as a 2 × 4 ANOVA with group (deaf, hearing) as between‐group factor and equation type (addition, subtraction, multiplication, division) as within‐subject factor. Results from this test could be important in order to interpret imaging results. For example, if deaf individuals had performed as fast as hearing individuals in multiplication but still engaged the right intraparietal sulcus, it would have indicated qualitatively different processes.

#### 2.5.3 In‐scanner responses

Results from the in‐scanner performance were analyzed to assess potential differences between groups and between the different equation types and difficulty levels. Response time and accuracy were analyzed in two separate mixed 2 × 2 × 2 analyses of variance with group (deaf or hearing) as a between‐subject factor and equation type (subtraction or multiplication) and difficulty (simple or difficult) as within‐subject factors. Due to technical problems, data from one deaf participant was lost.

## 3 RESULTS

### 3.1 Behavioral analyses

#### 3.1.1 In scanner results

**FIGURE 3:**
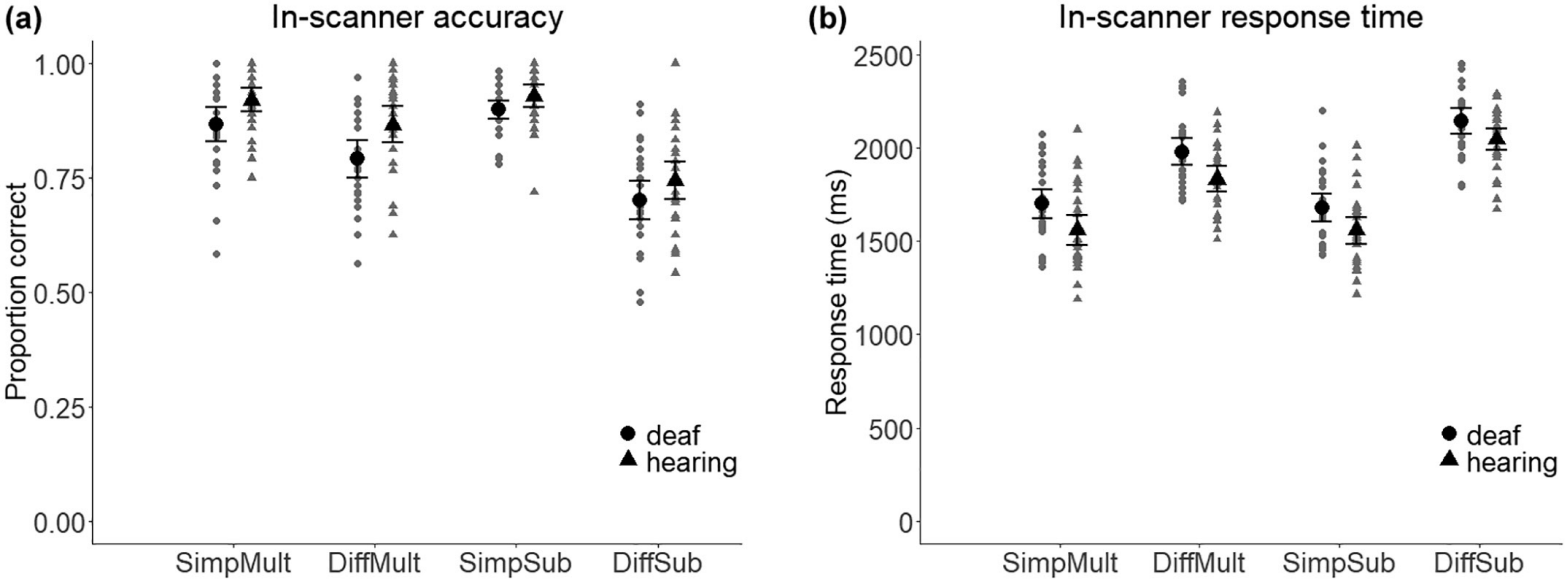
(a) Accuracy and (b) response time for in‐scanner behavioral measures. Error bars represent 95% confidence intervals. SimpMult, simple multiplication; DiffMult, difficult multiplication; SimpSub, simple subtraction; and DiffSubb, difficult subtraction.

Accuracy and response time data and group comparison data from the tasks performed during scanning are presented in Figure 3 and Table S1A For accuracy, the 2 × 2 × 2 analyses of variance showed a significant main effect of equation type, with better, *F*(1,55) = 23.8, *p* < .001, $\eta_{p}^{2}$ = 0.302 and faster, *F*(1,55) = 29.5, *p* < .001, $\eta_{p}^{2}$ = 0.349, performance on multiplication compared to subtraction. There was also a significant main effect of difficulty for both accuracy, *F*(1,55) = 180, *p* < .001, $\eta_{p}^{2}$ = 0.77, and response time, *F*(1,55) = 339, *p* < .001, $\eta_{p}^{2}$ = 0.86, with better and faster performance for simple compared to difficult tasks. Further, there was a significant main effect of group with hearing non‐signers performing better, *F*(1,55) = 7.07, *p* = .010, $\eta_{p}^{2}$ = 0.114, and faster, *F*(1,55) = 10.4, *p* = .002, $\eta_{p}^{2}$ = 0.159, compared to deaf signers. There was also a significant interaction between equation type and difficulty on both accuracy, *F*(1,55) = 75.7, *p* < .001, $\eta_{p}^{2}$ × 0.579, and response time, *F*(1,55) = 48.4, *p* < .001, $\eta_{p}^{2}$ = 0.468. Analyses of simple main effects showed better and faster performance in simple tasks for both multiplication (*p* < .001) and subtraction (*p* < .001). For simple tasks, subtraction was performed with better accuracy compared to multiplication (*p* = .032); however, there was no difference in response time (*p* = .626). However, for difficult tasks, multiplication was performed with both better accuracy (*p* < .001) and faster response time (*p* < .001) compared to subtraction (see Table S1B for details). The remaining interaction effect were not significant (see Table S1B).

#### 3.1.2 Other behavioral tasks

##### 3.1.2.1 Arithmetic skills

The test of arithmetic skills was analyzed with a 2 × 4 analysis of variance, which showed a main effect of equation type, *F*(3,53) = 80.7, *p* < .001, $\eta_{p}^{2}$ = 0.60, where addition was performed better than subtraction, *p* < .001, and division, *p* < .001, and multiplication better than subtraction, *p* = .003, and division, *p* < .001, and subtraction better than division, *p* < .001. There was also a main effect of group, *F*(1,53) = 7.98, *p* = .007, $\eta_{p}^{2}$ = 0.13, with better performance in the hearing compared to deaf group. The performance was comparable across groups and arithmetic task as indicated by lack of significant interaction effect, *F*(3,53) = 0.243, *p* = .866, $\eta_{p}^{2}$ = 0.005. See Figure S1A.

##### 3.1.2.2 Working memory—Corsi blocks

There were no differences between groups on the spatial working memory test, Corsi blocks, *t*(55) = 0.478, *p* = .634, *r* = 0.06. The mean span length for deaf signers in was 4.46 (SD = 1.23) and in hearing non‐signers, it was 4.62 (SD = 1.24).

### 3.2 Whole‐brain analyses

**FIGURE 4:**
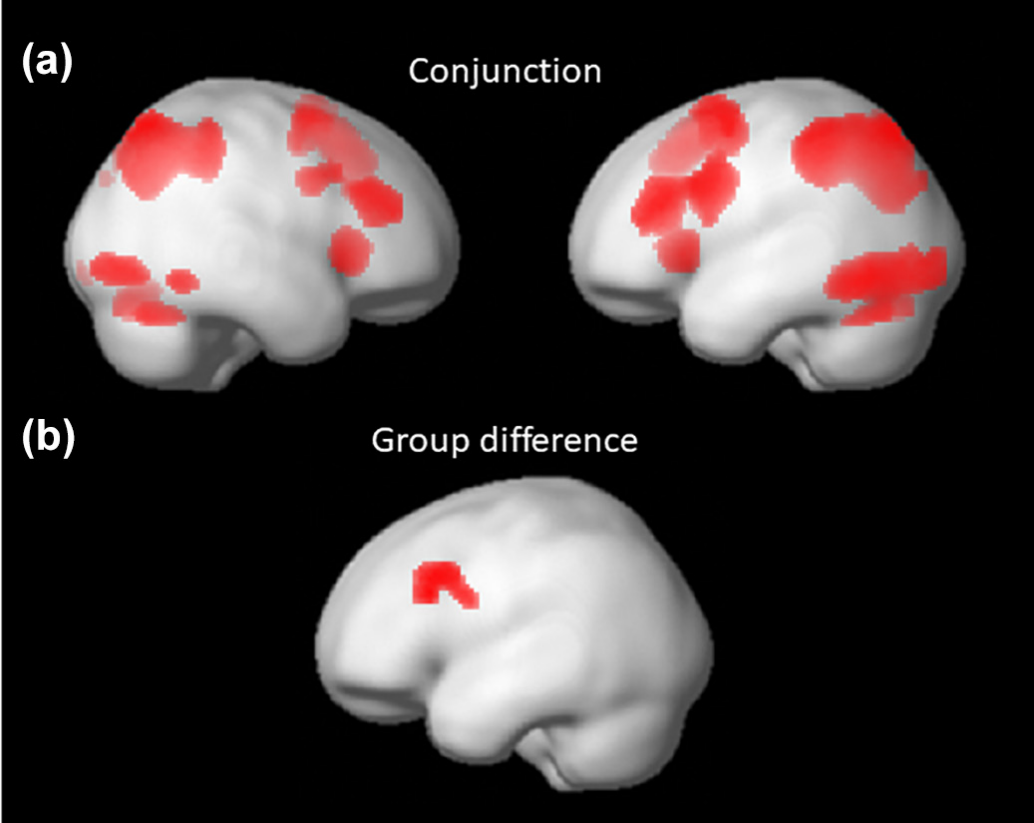
Significant activation for (a) conjunction analysis across tasks and groups (displayed at FEW‐corrected *p* < .05 at peak level) and (b) hearing non‐signers > deaf signers (displayed at uncorrected *p* < .001 at peak level with an extent threshold of 50 voxels).

There was a large overlap in activation across deaf and hearing individuals as indicated by conjunction analysis across groups (Figure 4a, Table 3). This conjunction analysis showed consistently high and jointly significant activation across tasks and groups in a widespread bilateral fronto‐parietal network, including inferior and superior parietal areas, inferior and middle frontal areas, and insula. There was also joint activation in occipital areas and the cerebellum.

**TABLE 3:** Peak and cluster activation for conjunction analysis across groups and tasks

Effect	Cluster level	Peak level		*x*	*y*	*z*	Region
	*p*_fwe_	*p*_fwe_	*T*				
Conjunction	<.001	<.001	12.1	−24	−68	48	l. superior parietal lobule
		<.001	10.1	−28	−54	48	l. inferior parietal lobule
		<.001	9.93	−40	−44	48	l. inferior parietal lobule
	<.001	<.001	10.8	−40	6	32	l. precentral gyrus
		<.001	9.40	−42	30	22	l. inferior frontal gyrus (p. triangularis)
		<.001	8.91	−30	24	0	l. insula lobe
	<.001	<.001	9.50	−50	−60	−12	l. inferior temporal gyrus
		<.001	7.17	−28	−64	−28	l. cerebelum VI
		<.001	5.95	−36	−88	−10	l. inferior occipital gyrus
	<.001	<.001	9.21	32	24	0	r. insula lobe
	<.001	<.001	9.17	−6	18	48	l. posterior‐medial frontal
		<.001	9.02	4	16	52	r. posterior‐medial frontal
		<.001	8.81	−26	2	56	l. middle frontal gyrus
	<.001	<.001	8.48	24	−62	52	r. superior parietal lobule
		<.001	7.81	30	−72	38	r. middle occipital gyrus
		<.001	7.60	40	−42	46	r. inferior parietal lobule
	<.001	<.001	8.29	30	−64	−26	r. cerebelum VI
		<.001	7.38	8	−76	−22	r. cerebelum VI
		<.001	7.21	−4	−76	−24	l. cerebelum crus 1
	<.001	<.001	6.93	32	6	64	r. superior frontal gyrus
	<.001	<.001	6.62	46	32	24	r. inferior parietal gyrus (p. triangularis)
		<.001	6.04	46	10	36	r. inferior frontal gyrus (p. opercularis)
		<.001	5.87	50	44	20	r. middle frontal gyrus
	<.001	<.001	6.20	42	−80	−6	r. inferior occipital gyrus
		<.001	6.16	36	−86	−6	r. inferior occipital gyrus
	.001	<.001	6.10	56	−52	−12	r. inferior Temporal gyrus

The whole‐brain data were analyzed with a 2 × 2 × 2 analysis of variance with group (deaf, hearing) as a between‐subject factor, and equation type (multiplication, subtraction) and difficulty (simple, difficult) as within‐subject factors. The only region that showed a significant main effect of group at cluster level was a cluster encompassing the left inferior frontal gyrus and left precentral gyrus, with stronger activation in the hearing compared to the deaf group (Figure 4b, Table 4). At peak level, a peak in the left inferior frontal gyrus (pars opercularis), approached significance (*p*_fwe_ = .072). There was no significant interaction effect including group.

**TABLE 4:** Whole‐brain peak and cluster activation for all significant main and interaction effects and simple main effects pertaining to the significant interaction effect

Effect	Comparison	Cluster level	Peak level					
		*p*_fwe_	*p*_fwe_	*F*	*x*	*y*	*z*	Region
Group	Hearing non‐signers > deaf signers^a^	.040	.072	21.2	−52	22	34	l. inferior frontal gyrus (p. Opercularis)
			.355	16.9	−48	2	24	l. precentral gyrus
			.783	13.8	−46	20	26	l. inferior frontal gyrus (p. Triangularis)
Equation type	Subtraction > multiplication^a^	<.001	<.001	82.5	10	−64	56	r. precuneus
			<.001	48.1	−8	−60	60	l. precuneus
			<.001	34.9	8	−50	46	r. precuneus
		<.001	<.001	64.5	42	−76	26	r. middle occipital gyrus
		<.001	<.001	45.5	26	4	58	r. superior frontal gyrus
		<.001	<.001	39.2	56	−46	38	r. inferior parietal lobule
			<.001	35.3	32	−42	44	r. intraparietal sulcus (hIPS3)
			.001	30.8	44	−34	44	r. supramarginal gyrus
		<.001	<.001	32.3	−28	−4	56	l. precentral gyrus
			.003	29.1	−18	−2	58	
		.001	.002	29.8	−34	−84	32	l. middle occipital gyrus
		.003	.004	28.4	−66	−40	28	l. intraparietal lobule (angular gyrus)
		.022	.028	23.6	36	40	36	r. middle frontal gyrus
		.042	.028	23.6	−54	−72	−2	l. middle occipital gyrus
		.031	.035	23.0	58	−64	−4	r. inferior temporal gyrus
Difficulty	Simple > difficult	<.001	<.001	64.2	−50	−66	40	l. angular gyrus
			<.001	59.0	−46	−60	28	l. angular gyrus
		<.001	<.001	59.3	−8	−50	32	l. posterior cingulate cortex
			<.001	55.7	8	−54	32	r. precuneus
		<.001	<.001	58.3	56	−62	34	r. angular gyrus
			<.001	42.8	48	−66	48	r. angular gyrus
		<.001	<.001	42.7	0	60	−4	l. middle orbital gyrus
			<.001	35.0	−8	60	22	l. superior medial gyrus
			<.001	34.5	−16	36	56	l. superior frontal gyrus
		.009	.003	29.1	64	−54	6	r. middle temporal gyrus
		.005	.008	26.8	−60	−12	−24	l. middle temporal gyrus
		.034	.025	23.9	2	32	−14	l. mid orbital gyrus
		.042	.047	22.3	−50	30	−10	l. inferior frontal gyrus (p. orbitalis)
	Difficult > simple	<.001	<.001	99.5	34	24	0	r. insula
			<.001	47.3	38	34	22	r. middle frontal gyrus
			.006	27.4	36	42	36	r. middle frontal gyrus
		<.001	<.001	77.8	−30	22	0	l. insula
			<.001	43.4	−42	28	24	l. inferior frontal gyurs (p. triangularis)
			<.001	42.8	−44	6	30	l. precentral gyrus
		<.001	<.001	72.8	4	20	48	r. posterior‐medial frontal
			<.001	38.3	10	28	30	r. midcingulate cortex
		<.001	<.001	68.3	−22	6	56	l. middle frontal gyrus
		<.001	<.001	65.8	−38	−84	−10	l. inferior occipital gyrus
			<.001	56.2	−48	−64	−10	l. inferior temporal gyrus
			<.001	41.3	−28	−64	−28	l. cerebelum VI
		<.001	<.001	65.8	32	−90	−6	r. inferior occipital gyrus
		<.001	<.001	58.9	−22	−66	42	l. superior parietal lobule
			<.001	54.5	−28	−52	50	l. superior parietal lobule
			<.001	47.1	−42	−42	46	l. inferior parietal lobule
		<.001	<.001	49.1	26	6	60	r. superior frontal gyrus
			<.001	41.5	32	−4	48	r. precentral gyrus
		<.001	<.001	47.0	28	−46	42	
			<.001	40.9	42	−42	44	r. inferior parietal lobule
			<.001	36.4	30	−60	52	r. angular gyrus
		<.001	<.001	45.2	−4	−74	−24	l. cerebelum crus 1
			<.001	39.6	6	−74	−24	cerebellar vermis 7
			.017	24.8	2	−60	−24	cerebellar vermis 6
		.004	<.001	32.3	28	−64	−26	r. cerebelum VI
		.042	.035	23.0	22	42	−10	
Type × difficulty		.001	.005	4.99	−42	−80	32	l. middle occipital gyrus
		.012	.007	4.90	38	−78	38	r. inferior parietal lobule
		.025	.028	4.58	−60	−2	8	l. rolandic operculum
Simple main effects								
Subtraction: simple > difficult		.028	.017	4.69	−60	−2	8	l. rolandic operculum
Subraction: difficult > simple		.004	<.001	6.27	−32	−78	40	l. middle occipital gyrus
			<.001	6.21	−34	−84	34	l. middle occipital gyrus
		.020	<.001	5.55	36	−78	38	r. middle occipital gyrus
Difficult: subtraction > multiplication		.013	<.001	7.03	38	−78	36	r. middle occipital gyrus
		.001	<.001	6.90	−36	−84	34	l. middle occipital gyrus

*Note*: The table shows activation peaks and clusters for each main effect, FWE‐corrected at *p* < .05 for all comparisons except the main effect of group, which is thresholded at uncorrected *p* < .001 with an extent threshold of 50 voxels. Brain regions are based on the cytoarchitectonic probability maps of Anatomy Toolbox in SPM12.

^a^ Theres were no significant effects for the opposite contrast.

For the main effect of equation type, there were widespread activations across bilateral parietal areas; however, with a right‐lateralized bias (Table 4, Figure S2A). There were also some activation clusters in the frontal and temporal regions. All significant clusters showed stronger activation for subtraction compared to multiplication.

Activation for the main effect of difficulty was found in widespread fronto‐parietal regions in both hemispheres for difficult compared to simple tasks (Table 4, Figure S2B). For simple compared to difficult tasks, activation was present mainly in the bilateral angular gyrus, posterior cingulate cortex, and superior frontal and orbital regions.

We also found a significant interaction effect between the equation type and difficulty in the left middle occipital gyrus, right inferior parietal lobule, and left Rolandic operculum (Table 4, Figure S2C). Further investigations of the interaction effect showed that for subtraction, simple tasks showed stronger activation than difficult tasks in the left Rolandic operculum, while the opposite contrast showed activation in the left occipital gyrus. Finally, for difficult tasks, subtraction elicited stronger activation than multiplication in the middle occipital gyrus.

### 3.3 Region‐of‐interest analyses

**FIGURE 5:**
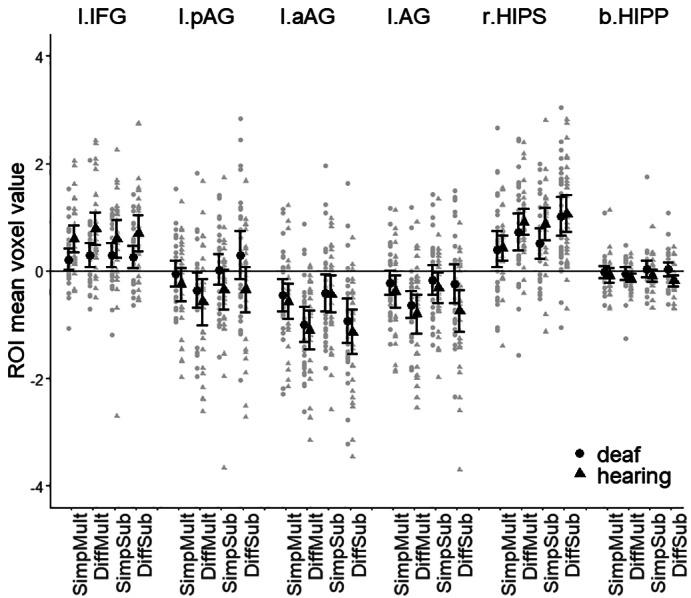
Activation within different ROIs. ROI mean values are presented for each condition. Error bars represent 95% confidence intervals. b.Hipp, bilateral hippocampus; DiffMult, difficult multiplication; DiffSubb, difficult subtraction; l.aAG, left anterior angular gyrus; l.AG, left angular gyrus (both posterior and anterior portions combined); L.IFG, left inferior frontal gyrus; l.pAG, left posterior angular gyrus; rHIPS right horizontal portion of the intraparietal sulcus; SimpMult, simple multiplication; SimpSub, simple subtraction.

To investigate predictions related to specific regions, region‐of‐interest analyses were performed separately for the anterior and posterior portion of the left angular gyrus, the whole angular gyrus, the right horizontal intraparietal sulcus, the left inferior frontal gyrus, and the bilateral hippocampus (Figure 5).

#### 3.3.1 Left inferior frontal gyrus

The left inferior frontal gyrus was significantly activated in all tasks in both groups. The 2 × 2 × 2 analysis of variance showed a significant main effect of group, *F*(1,56) = 6.786, *p* = .012, $\eta_{p}^{2}$ = 0.108, with stronger activation for the hearing compared to the deaf group. No other main or interaction effects were significant (all *p*:s > .117, Table S2). Hence, as predicted, there was significant activation for all tasks in the hearing group and multiplication in the deaf group. However, contrary to the predictions, there was also significant activation for subtraction in the deaf group. Further, as predicted, there was a generally stronger activation in the hearing group.

#### 3.3.2 Left posterior angular gyrus

In the posterior part of left angular gyrus, a significant deactivation was found for difficult multiplication for both groups. No activation or deactivation was found for the other conditions. In this region, there was a significant effect of equation type, *F*(1,56) = 12.242, *p* < .001, $\eta_{p}^{2}$ = 0.179, with stronger relative deactivation for multiplication compared to subtraction. The deaf group showed slightly less deactivation compared to the hearing group, but this main effect of group did not reach significance, *F*(1,56) = 3.135, *p* = .082, $\eta_{p}^{2}$ = 0.053. There was further a significant interaction effect between equation type and difficulty, *F*(1,56) = 13.049, *p* < .001, $\eta_{p}^{2}$ = 0.189. Investigation of the interaction effect showed that for multiplication, difficult compared to simple tasks resulted in stronger deactivation, *F* = 16.977, *p* < .001, $\eta_{p}^{2}$ = 0.233, whereas no such effect was present for subtraction, *F* = 1.311, *p* = .257, $\eta_{p}^{2}$ = 0.023. For difficult tasks, there was stronger deactivation for multiplication compared to subtraction, *F* = 20.344, *p* < .001, $\eta_{p}^{2}$ = 0.266. No such effect was found for simple tasks, *F* = 0.023, *p* = .880, $\eta_{p}^{2}$ < 0.001. No other main or interaction effects were significant (*p*:s > .134, Table S2). To sum up, the analyses revealed specific deactivation for difficult multiplication tasks, which was partly in line with the predictions, but no significant differences were found between groups.

#### 3.3.3 Left anterior angular gyrus

The anterior left angular gyrus was significantly deactivated for all tasks in both groups. The main effect of difficulty was significant, *F*(1,56) = 21.747, *p* < .001, $\eta_{p}^{2}$ = 0.484, with difficult compared to simple tasks being more strongly deactivated. No other main or interaction effects were significant (*p*:s < .310).

#### 3.3.4 Left angular gyrus

When the anterior and posterior left angular gyrus were analyzed as one region, the multiplication conditions were significantly deactivated in both groups and the subtraction conditions specifically in the hearing group. There was a significant, but small, main effect of equation type, *F*(1,56) = 4.498, *p* = .038, $\eta_{p}^{2}$ = 0.074, with stronger deactivation for multiplication compared to subtraction. There was also a significant main effect of difficulty, *F*(1,56) = 21.469, *p* < .001, $\eta_{p}^{2}$ = 0.277, with difficult tasks showing stronger deactivation compared to simple tasks. Finally, there was a significant, but small, interaction effect between equation type and difficulty, *F*(1,56) = 4.308, *p* = .043, $\eta_{p}^{2}$ = 0.071. Analyses of the interaction effect showed that for multiplication, the difficult condition led to significantly stronger deactivation, *F*(1,56) = 31.590, *p* < .001, $\eta_{p}^{2}$ = 0.361, whereas no such effect was found for subtraction, *F*(1,56) = 2.514, *p* = .118, $\eta_{p}^{2}$ = 0.043. For difficult tasks, there was significantly stronger deactivation for multiplication compared to subtraction, *F*(1,56) = 7.175, *p* = .010, $\eta_{p}^{2}$ = 0.114, whereas no significant difference was found for the simple conditions, *F*(1,56) = 0.0002, *p* = .988, $\eta_{p}^{2}$ < 0.001. Hence, this region was primarily deactivated rather than activated, as suggested in the predictions. However, as predicted, there was a difference from baseline (albeit deactivation) for multiplication, but not for subtraction, in the deaf group and for all tasks in the hearing group. However, contrary to our predictions, there were no group differences in the left angular gyrus.

#### 3.3.5 Right horizontal intraparietal sulcus

In the right horizontal intraparietal sulcus, all conditions for both groups showed significant activation from baseline. There was a significant main effect of equation type, *F*(1,56) = 15.167, *p* < .001, $\eta_{p}^{2}$ = 0.213, with stronger activation for subtraction compared to multiplication. The main effect of difficulty was also significant, *F*(1,56) = 17.854, *p* < .001, $\eta_{p}^{2}$ = 0.242, with stronger activation for difficult compared to simple conditions. No other main or interaction effects were significant (*p*:s > .186, Table S2). Hence, as predicted, there was significant activation in the right horizontal intraparietal sulcus for all tasks in the deaf group and difficult tasks in the hearing group. However, contrary to prediction, there was significant activation also for the simple tasks in the hearing group, but no group differences were found in this region.

#### 3.3.6 Bilateral hippocampus

In the bilateral hippocampus, the difficult conditions differed slightly from baseline in the hearing group. However, no main or interaction effects were significant (*p*:s > .114, Table S2). Contrary to our predictions, the bilateral hippocampus was not activated across conditions in any of the groups, and there was no group difference.

### 3.4 Connectivity analyses

Functional connectivity analyses performed across all tasks showed a multitude of group‐specific significant connections from the five seeds to the rest of the brain (Table S3). Differences between groups were found with lIFG as seed‐ROI, with stronger functional connectivity for deaf signers compared to hearing non‐signers to the left planum temporale, left superior temporal gyrus, and left planum polare (Table 5). When each equation type was analyzed separately, no significant differences were found.

**TABLE 5:** Group differences in functional connectivity

Seed‐ROI	Target	Deaf signers		Hearing non‐signers		Group differences	
		*Beta*	*p*	*Beta*	*p*	*t*	*p*
LIFG	Left planum temporale	0.31	<.001	0.13	.001	4.31	.003
	Left superior temporal gyrus (posterior)	0.41	<.001	0.24	<.001	3.64	.017
	Left planum polare	0.28	<.001	0.10	.021	3.65	.046

**FIGURE 6:**
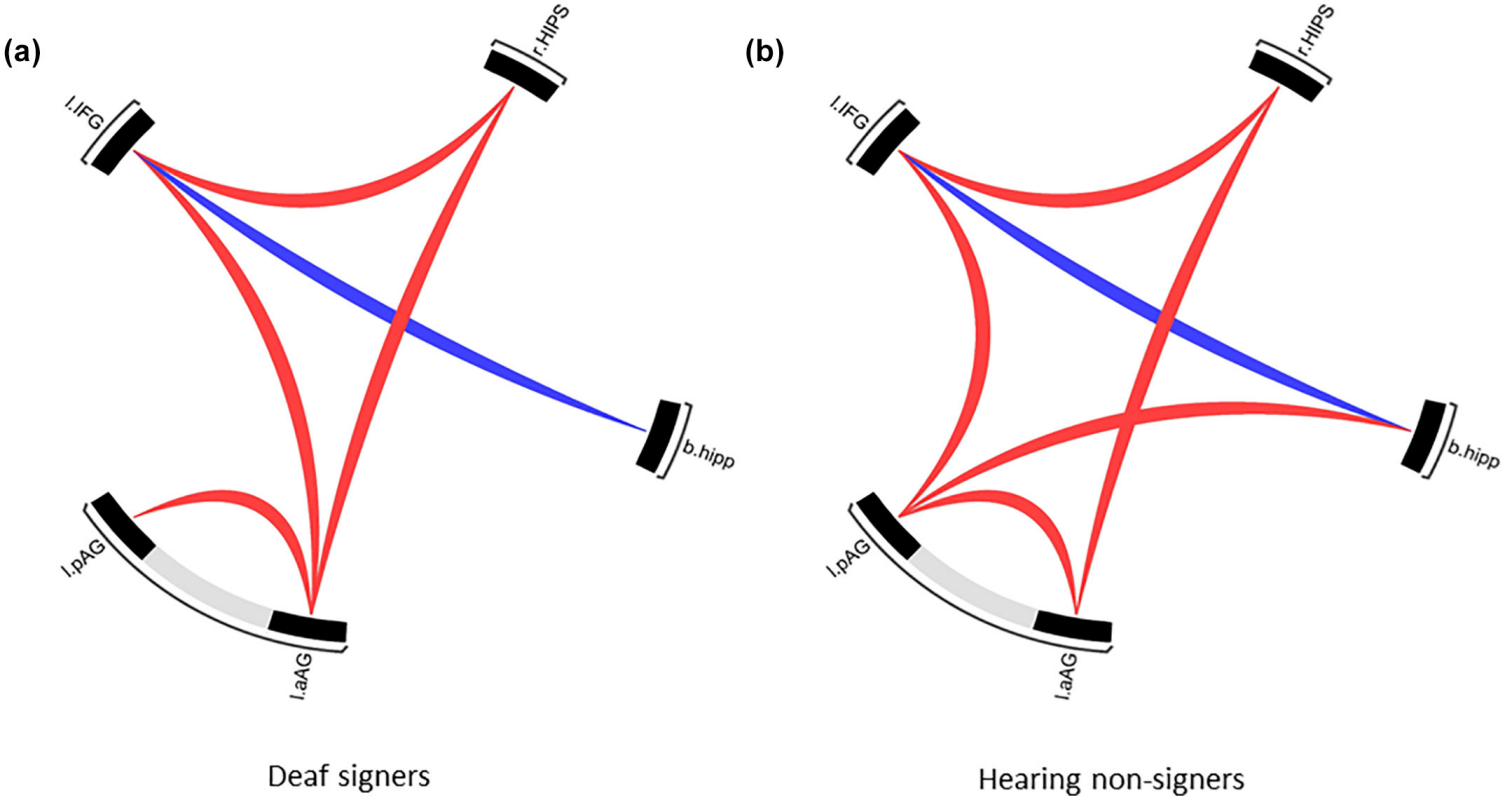
Functional connectivity between the left inferior frontal gyrus, left posterior and anterior angular gyrus, right horizontal intraparietal sulcus, and hippocampus in (a) deaf signers and (b) hearing non‐signers. The figure is a graphical representation of functional connectivity, with red lines representing significant positive correlation coefficients between the two given regions and blue representing significant negative correlations. b.hipp, bilateral hippocampus; l.aAG, left anterior angular gyrus; L.IFG, left inferior frontal gyrus; l.pAG, left posterior angular gyrus; r.HIPS right horizontal portion of the intraparietal sulcus.

Limiting the search area to the selected ROIs showed significant positive connectivity between the right horizontal intraparietal sulcus, left anterior angular, and left inferior frontal gyrus and negative connectivity between the hippocampus and left inferior frontal gyrus in both groups (Figure 6). Both groups also had significant positive connectivity between the anterior and posterior angular and between the left angular and left inferior frontal gyrus; however, for the deaf group, this connection was to the anterior angular gyrus, while for the hearing group, it was to the posterior angular gyrus. For the hearing group, there was also significant positive connectivity between the left posterior angular gyrus and hippocampus. For correlation coefficients, see Table S4.

### 3.5 Exploratory analyses

Both the whole brain and the ROI analyses showed significantly stronger activation in the hearing group in the left inferior frontal gyrus. Considering that the hearing group also performed both better and faster on the in‐scanner tasks as well as on the arithmetic skills test, a tentative interpretation could be that the activation differences are an effect of general arithmetic ability. To test this, we correlated ROI mean values in the left inferior frontal gyrus and in‐scanner performance, testing the post hoc hypothesis that this region is performance‐dependent. However, we found no significant correlations between ROI mean values and performance for all participants combined (simple multiplication *r* = 0.027, *p* = .839, difficult multiplication *r* = 0.227, *p* = .089, simple subtraction *r* = 0.071, *p* = .598, difficult subtraction *r* = 0.130, *p* = .335) or the groups separately (Table S5). For hearing non‐signers, the correlation for simple multiplication approached significance, however, the correlation was negative, *r* = −0.349, *p* = .063, such that better performance corresponded to lower ROI values. We also ran voxel‐wise brain‐behavioral correlation analyses and failed to find any significant relationship between task and activation within the left inferior frontal gyrus (for difficult subtraction we found a peak at −56 18 26 in the hearing group that was significant at uncorrected *p* = .001, but after fwe‐correction *p* = .993). Hence, there was no support for the notion that group differences in the left inferior frontal gyrus reflect performance differences.

## 4 DISCUSSION

**FIGURE 7:**
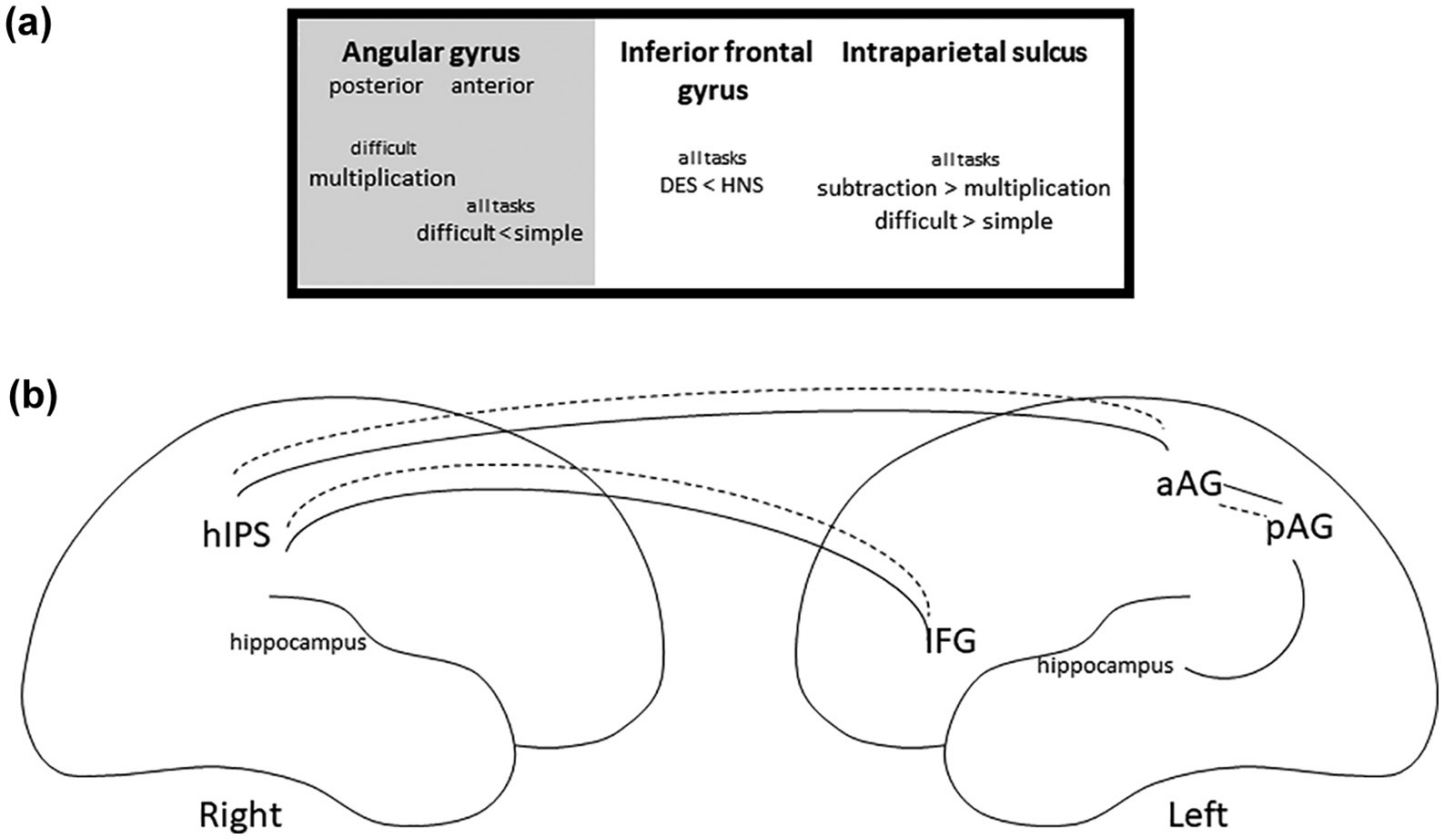
In contrast to the predictions outlined in Figure 1, we found relatively few differences between groups both in the recruitment of specific regions and in terms of functional connectivity. (a) in the posterior angular gyrus, the only significant activation found was for difficult multiplication compared to baseline. In the anterior angular gyrus, all tasks showed an effect of difficulty. In the inferior frontal gyrus, there was an effect of group across all tasks and in the intraparietal sulcus subtraction showed stronger activation than multiplication and difficult tasks stronger activation than simple task. (b) Straight lines indicate significant functional connectivity in hearing individuals and dotted line in deaf individuals. aAG, anterior angular gyrus; hIPS, horizontal portion of the intraparietal sulcus; IFG, inferior frontal gyrus; pAG, posterior angular gyrus.

In the present study, we investigated the neural correlates behind arithmetic processing in deaf signing adults. Generally, we found overlapping patterns of activation across deaf and hearing individuals in a widespread bilateral fronto‐parietal network. The left inferior frontal gyrus was the only region where we could identify differences in activation between the groups, that is, stronger activation for the hearing compared to the deaf group was found in both whole‐brain and ROI analysis. We also found differences in functional connectivity between the left inferior frontal gyrus and left superior temporal gyrus, where the deaf group had stronger connectivity. Contrary to our predictions, we found no support for group differences in the left angular gyrus, right horizontal intraparietal sulcus, or hippocampus. We conclude that group differences were restricted to the left inferior frontal gyrus and its communication with the left superior temporal gyrus in arithmetic and that we did not find support for qualitatively different engagement of verbal versus magnitude processes between groups. Hence, although we found support for parts of our predictions, the outcome overall tells a different story than that we had expected (compare predictions in Figure 1 and outcome in Figure 7).

The only significant group difference in the brain imaging data was the predicted stronger activation of the left inferior frontal gyrus in the hearing group. This pattern was found in both the ROI and whole‐brain analyses. In addition, the deaf participants exhibited significantly poorer performance on the arithmetic tasks. Two different explanations could potentially explain these patterns of results.

The first potential explanation is that lower activation in general cognitive processing within the left inferior frontal gyrus is associated with lower arithmetic performance in the deaf group. The left inferior frontal gyrus is associated with both domain‐general cognitive processes and language‐specific properties. Some studies have found that arithmetic tasks are processed mainly in the domain‐general periphery of the left inferior frontal gyrus (e.g., Fedorenko et al., 2012), suggesting that this region might mainly be involved in arithmetic through its dependence on general cognitive ability (not through language ability). Stronger activation for difficult compared to simple tasks in the region corroborates this line of reasoning (Artemenko et al., 2018; e.g., Fedorenko et al., 2012; Soltanlou et al., 2017). In the present study, we found a significant peak for difficult compared to simple tasks in the periphery of the left inferior frontal gyrus in the whole‐brain analysis. These results support the hypothesis that this region contributes to arithmetic processing through its domain‐general, rather than language‐specific properties. The peaks from the stronger activated cluster in the hearing compared to the deaf group (Table 4) in the outer parts of the region are also in accordance with the idea of domain‐general processing related to arithmetic rather than weaker general activation in the verbal system for the deaf group. Although activation in the left inferior frontal gyrus was significant in both the hearing and deaf groups, it was stronger for the hearing group, suggesting that the difference might stem from the generally weaker performance in the deaf group. This is also supported by results from our previous study where a lack of performance difference was accompanied by no significant differences in activation (Andin et al., 2019). However, the exploratory correlation analysis showed no significant correlation between performance and activation across groups or within groups, leaving this hypothesis unsupported (exploratory analyses).

The second potential explanation for the weaker activation of domain‐general regions is a cross‐modal functional shift in the deaf participants. In deaf individuals, the auditory cortex is deprived of sensory input during development which leads to cross‐modal plastic changes probably including both functional preservation, that is, the deprived sensory region responds to different sensory input, and functional shift, that is, the deprived region reorganizes to respond to higher‐order cognitive tasks (Cardin et al., 2020). Thus, it has been shown that the auditory cortex, in particular the superior temporal gyrus, responds to working memory (Cardin et al., 2013; Ding et al., 2015; Twomey et al., 2017) and that this region can be an additional resource during cognitive processing. In the present study, we found significantly stronger functional connectivity between the left inferior frontal gyrus and the left superior temporal gyrus, which might indicate a role for the superior temporal gyrus in domain‐general processing of arithmetic tasks in deaf individuals. However, this does not explain differences in arithmetic performance. Further studies are needed to improve our understanding of the role of the superior temporal gyrus for arithmetic processing in deaf individuals.

The original triple code model suggested the left angular gyrus as a node for verbal processing of numbers, in general, and specifically arithmetic (Dehaene et al., 2003). Recent studies have challenged the model and suggested a more domain‐general role of this region (Bloechle et al., 2016; Uddin et al., 2010), at least for the posterior part. Wu et al. (2009) found the posterior part to be strongly deactivated during arithmetic while the anterior part was less deactivated (although not activated above baseline). The results from the present study showed mainly deactivation in both the posterior and anterior parts; however, the pattern was the opposite: we found significant deactivation for all tasks in the anterior part, but only for the difficult multiplication in the posterior part.

The posterior angular gyrus is connected to the default mode network, such that a deactivation occurs when attention shifts from rest to task. The degree of deactivation may depend on the difficulty of the task (Uddin et al., 2010). The interaction effect between difficulty and equation type showed stronger deactivation in difficult compared to simple tasks for multiplication, but not for subtraction. We suggest that this reflects specific allocation of attention during verbal fact retrieval that is required during multiplication. Further, although the group comparison was not significant (*p* = .082), the deaf group had slightly stronger absolute activation in this part (less deactivation), suggesting less allocation of attention for verbal fact retrieval. This, together with the generally lower activation in the left inferior frontal gyrus, might indicate that this group uses other strategies than verbal fact retrieval to a higher degree in arithmetic. Further evidence for this notion comes from the connectivity analyses, where the connectivity between the posterior angular gyrus and left inferior frontal gyrus, corroborating Uddin et al. (2010), was significant for the hearing, but not the deaf, individuals (although no significant group difference).

In contrast to previous studies, the anterior angular gyrus showed a more remarked deactivation than the posterior part across all tasks and both groups. There was a clear difficulty modulation with stronger deactivation for difficult tasks. Wu et al. (2009) used rather simple combinations of addition and subtraction and found no significant change from baseline in the left anterior angular gyrus, while there was a deactivation in the posterior part. Given the clear difficulty effect in the present study, it is possible that differences in task difficulty between theirs and our study can explain these inconsistencies. Interestingly, the anterior angular gyrus showed significant functional connectivity with the left inferior frontal gyrus for the deaf group. Hence, while the angular gyrus and left inferior frontal gyrus were functionally connected via the posterior part in hearing individuals, the connection for deaf individuals is to the anterior part, potentially reflecting different verbal processing networks. It should, however, be pointed out that these connections did not differ significantly between groups.

In sum, the results from the present study supports recent previous studies of deactivation rather than activation in the angular gyrus during arithmetic processing and that the same pattern was found for deaf signers, at least in the anterior part. Contrary to suggestions of a stronger involvement of anterior compared to posterior angular gyrus for arithmetic processing (Uddin et al., 2010; Wu et al., 2009), we found the opposite. Our results suggest that the posterior angular gyrus might be involved in allocation of attention, however, especially connected to verbal fact retrieval in multiplication. While the anterior part showed a more general difficulty modulation effect across both multiplication and subtraction. Most importantly, we found no differences between groups.

Recent studies have suggested that the hippocampus, rather than the left angular gyrus, is responsible for fact retrieval during arithmetic processing (Bloechle et al., 2016; Klein et al., 2016; for a review see Menon, 2010). Our ROI results showed no clear effects in the hippocampus lending little support for this hypothesis. However, as predicted, we found significant functional connectivity between the hippocampus and posterior angular gyrus in the hearing group and a tendency towards significant connectivity in the deaf group (*p* = .089). In combination with the lack of connection between the right horizontal intraparietal sulcus and hippocampus, the results support a general interplay between the posterior angular gyrus and hippocampus during arithmetic processing, at least in hearing individuals.

The predicted role of the right horizontal intraparietal sulcus is magnitude processing along the mental number line, a process that is expected to take place when automatic fact retrieval is not enough, for example, during subtraction and difficult tasks. In line with this prediction, we found the right horizontal intraparietal sulcus to be more strongly recruited during subtraction compared to multiplication and difficult compared to simple tasks. However, these effects were similar across groups; hence, we did not find stronger activation in the deaf compared to the hearing group. This corroborates findings in the recent study by Berteletti et al. (2022), who used event‐related potentials to show the same attentional patterns for simple arithmetic problems in both deaf signing and hearing non‐signing adults as well as the same attentional dissociation for subtraction and multiplication. Further, we found all tasks to activate the right horizontal intraparietal sulcus significantly in both groups. The role of this region as central to magnitude processing is, hence, contested by the significant activation of simple multiplication we found. There are different explanations for this finding. Skagenholt (2022) suggest that activation found during number processing in the intraparietal sulcus reflects attentional and working memory load rather than magnitude processing. Another explanation could be that the design of the present study, with alternating blocks of different task types, bias participant to rely on quantity‐based strategies with sustained activation of the intraparietal sulcus. The present results further contrasts with our previous findings of significantly stronger activation in deaf compared to hearing individuals for simple multiplication (Andin et al., 2019) and lends no support for the prediction that lower reliance on verbal fact retrieval in the deaf group would lead to stronger activation in the right horizontal intraparietal sulcus, especially for simple multiplication. In our previous study, the deaf individuals performed equally to the hearing individuals on multiplication, which indicate that the stronger recruitment of the right horizontal intraparietal sulcus in that study might be an effect of effective compensatory activation to support lower reliance on verbal strategies. Soltanlou et al. (2022) suggested that given similar performance in an atypical and typical group, higher mental effort in the atypical group would be expected to relate to higher brain activity. However, given similar activation in the two groups, lower performance would be expected in the atypical group. In this study, the group of deaf individuals performed at a lower level on arithmetic compared to the hearing group. Along the same line of reasoning this pattern could indicate a failure to engage in the compensatory mechanisms that activation in the right intraparietal sulcus could provide.

This might further indicate that the deaf individuals in the present study have not acquired such effective compensatory mechanisms. Contrary to our prediction that only deaf individuals would show functional connectivity between the left inferior frontal gyrus and right horizontal intraparietal sulcus, we found this to be true for both groups, although with a slightly higher beta in the deaf group (0.15) compared to the hearing (0.096). Skagerlund et al. (2019) investigated functional connectivity between these regions and found stronger connectivity with high arithmetic proficiency, which was not the case here. Together with the direction of the group difference in the left inferior frontal gyrus, it appears that patterns found in hearing individuals do not transfer to deaf individuals; deaf individuals show lower activity in the left inferior frontal gyrus and equal (or slightly higher) functional connectivity between the left inferior frontal gyrus and right horizontal intraparietal sulcus despite lower arithmetic performance.

There are some limitations in the present study. Based on our power calculations, we aimed to include 34 participants per group, but we had to stop at 29, which meant that we did not have 80% power to reach a significant group difference in the left inferior frontal gyrus (based on the statistical maps from Andin et al., 2019). However, that was the only region where we found group differences, hence, low power did not prevent us from finding group differences. This could be explained by differences in the design between the study used for power analysis (Andin et al., 2019) and the present study. It is also possible that the power analysis we did underestimated the power in other regions, such that the lack of effects in the present study is due to low power.

In our previous study, the work of Andin et al. (2019), we tested individuals from the same population, that is, young deaf signing adults, but did not find any group differences in arithmetic performance. This contrasts with the present study where the deaf group performed generally poorer across tasks in both the in‐scanner task and in the test of general arithmetic skills. The difference between groups might lead to differences at the neural level merely because of different skill levels. However, the lack of group differences in age, education, general cognitive abilities, and working memory, indicate that the group is generally on par with the hearing group and that the difficulties are limited to arithmetic processing, which is in line with the literature. We speculated that the high performance in Andin et al. (2019) reflected a generally better arithmetic development in the Swedish young deaf population due to optimized and equal linguistic development support. However, in the light of the present results that might have been an effect of skewed sampling.

In the present study, we adhered strictly to brain regions suggested to be involved in number processing in the original triple code model (Dehaene et al., 2003). However, several recent meta‐analyses and empirical articles have suggested other regions to be more important for number processing. Such regions include, for example, the right precuneus, left superior frontal gyrus, bilateral insula, and bilateral middle frontal gyrus (Hawes et al., 2019). Further analyses of these regions, as well as investigation of the possible specific involvement of the left superior temporal gyrus in deaf individuals, are needed to better understand arithmetic processing in deaf signers.

## 5 CONCLUSION

In the present study, we show that largely similar brain regions are engaged in arithmetic processing in deaf signers and hearing non‐signers. Regions generally associated with verbal processing of arithmetic fact retrieval and quantity processing along the mental number line were engaged across all tasks in both groups. Both the left angular gyrus and right horizontal intraparietal sulcus were sensitive to difficulty modulations, whereas the right horizontal intraparietal sulcus, as predicted, was also specifically sensitive to subtraction. The lack of differences in activation patterns, in particular, of the right intraparietal sulcus, in combination with generally poorer performance in the deaf compared to the hearing group might indicate a failure to recruit compensatory mechanisms. This contrasts to our previous study (Andin et al., 2019) in which we found activation differences in this region while performance was similar across groups. The only significant group differences we found in the present study were related to the left inferior frontal gyrus; deaf signers had lower activation across tasks and stronger functional connectivity to the left superior temporal gyrus, which could be related to generally poorer arithmetic performance in combination with cross‐modal functional shifts with larger involvement of temporal regions in deaf individuals. This may indicate differences in how verbal processing networks support arithmetic skills. In sum, we found no support for modality‐specific reliance on verbal versus magnitude systems as an explanation for poorer arithmetic performance. We suggest that explanations for performance differences might be found in other brain regions not included in the original triple code model.

### DECLARATION OF TRANSPARENCY

The authors, reviewers and editors affirm that in accordance to the policies set by the *Journal of Neuroscience Research*, this manuscript presents an accurate and transparent account of the study being reported and that all critical details describing the methods and results are present.

## AUTHOR CONTRIBUTIONS

**Josefine Andin**: Conceptualization; methodology; software; validation; formal analysis; investigation; data curation; writing ‐ original draft; visualization; supervision; project administration and funding acquisition. **Åsa Elwér**: Methodology; softeware; investigation; writing ‐ review and editing. **Elina Mäki‐Torkku**: Methodology; writing ‐ review and editing.

## CONFLICT OF INTEREST

No conflict of interest to declare.

### PEER REVIEW

The peer review history for this article is available at https://publons.com/publon/10.1002/jnr.25138

## Supporting information


**TABLE S1A** Accuracy and response time of the arithmetic in‐scanner tasks
**TABLE S1B** Main effects, interaction effects and simple main effects of the significant interaction effect (equation type × difficulty)
**TABLE S2** Main and interaction effects from the ROI analyses
**TABLE S3** Resting‐state functional connectivity results for all targets. Only connections with FDR‐corrected *p* < .001 are shown
**TABLE S4** Resting‐state functional connectivity results for only ROI targets
**TABLE S5** Correlation between performance and mean ROI values in left inferior frontal gyrus
**FIGURE S1** Arithmetic skills measured as number of correct answers within 2 min for the respective equation type. Error bars represent 95% confidence intercal.
**FIGURE S2** (a) Effect of equation type; green = subtraction > multiplication within the main effect of equation type contrast, (b) effect of difficulty; red = simple > difficult, green = difficult > simple within the main effect of equation type contrast, (c) simple main effects; red = subtraction: simple > difficult, green = subtraction difficult > simple, blue = difficulty subtraction > multiplication, turquoise = overlap between green and blue, within the interaction of type and difficulty.

Transparent Science Questionnaire for Authors

## Data Availability

Behavioural data: Data openly available at https://osf.io/cy4ag/ fMRI data: Data available on request due to privacy/ethical restrictions.

## Endnote

^1^
Power calculations based on 29 individuals per group yielded a power of 74%.
