# Supplementary material for: Arithmetic in the signing brain: Differences and similarities in arithmetic processing between deaf signers and hearing non‐signers
Source: J Neurosci Res. 2022 Oct 19;101(1):172–95. doi: 10.1002/jnr.25138 (PMC9828253; doi:10.1002/jnr.25138)
Supplement: Supplementary file 1 — TABLE S1A Accuracy and response time of the arithmetic in‐scanner tasks TABLE S1B Main effects, interaction effects and simple main effects of the significant interaction effect (equation type × difficulty) TABLE S2 Main and interaction effects from the ROI analyses TABLE S3 Resting‐state functional connectivity results for all targets. Only connections with FDR‐corrected p < .001 are shown TABLE S4 Resting‐state functional connectivity results for only ROI targets TABLE S5 Correlation between performance and mean ROI values in left inferior frontal gyrus FIGURE S1 Arithmetic skills measured as number of correct answers within 2 min for the respective equation type. Error bars represent 95% confidence intercal. FIGURE S2 (a) Effect of equation type; green = subtraction > multiplication within the main effect of equation type contrast, (b) effect of difficulty; red = simple > difficult, green = difficult > simple within the main effect of equation type contrast, (c) simple main effects; red = subtraction: simple > difficult, green = subtraction difficult > simple, blue = difficulty subtraction > multiplication, turquoise = overlap between green and blue, within the interaction of type and difficulty. [file JNR-101-172-s002.docx]

# Supplementary material

**Table S1A.** Accuracy and response time of the arithmetic in-scanner tasks.

|  |  |  | Deaf signers | |  | Hearing non-signers | |  |  |  |  |
| --- | --- | --- | --- | --- | --- | --- | --- | --- | --- | --- | --- |
| Measurement | Type | Difficulty | *m* | *sd* |  | *m* | *sd* |  | *t* | *p* | *r* |
| Accuracy | Multiplication | simple | 86.6 | 9.70 |  | 92 | 6.66 |  | 2.44 | 0.018 | 0.31 |
|  |  | difficult | 79.1 | 10.7 |  | 86.6 | 10.5 |  | 2.68 | 0.010 | 0.34 |
|  | Subtraction | simple | 89.9 | 5.24 |  | 92.9 | 6.36 |  | 1.99 | 0.052 | 0.26 |
|  |  | difficult | 70.0 | 10.9 |  | 74.4 | 10.9 |  | 1.51 | 0.136 | 0.19 |
|  | Baseline |  | 87.9 | 5.00 |  | 90.1 | 4.00 |  | 1.96 | 0.055 | 0.26 |
| Response time | Multiplication | simple | 1700 | 194 |  | 1560 | 216 |  | 2.58 | 0.013 | 0.33 |
|  |  | difficult | 1978 | 183 |  | 1832 | 179 |  | 3.03 | 0.004 | 0.38 |
|  | Subtraction | simple | 1680 | 197 |  | 1557 | 190 |  | 2.39 | 0.020 | 0.31 |
|  |  | difficult | 2144 | 174 |  | 2045 | 151 |  | 2.28 | 0.027 | 0.29 |
|  | Baseline |  | 1160 | 136 |  | 1155 | 166 |  | 0.138 | 0.891 | 0.02 |

**Table S1B.** Main effects, interaction effects and simple main effects of the significant interaction effect (Equation type x difficulty).

|  | Accuracy | | |  | Response time | | |
| --- | --- | --- | --- | --- | --- | --- | --- |
|  | *F* | *p* | *pη^2^* |  | *F* | *p* | *pη^2^* |
| ***Main effects*** |  |  |  |  |  |  |  |
| Group | 7.07 | .010 | .114 |  | 10.4 | .002 | .159 |
| Equation type | 23.8 | < .001 | .302 |  | 29.5 | < .001 | .349 |
| Difficulty | 180 | < .001 | .766 |  | 339 | < .001 | .860 |
| ***Interaction effects*** |  |  |  |  |  |  |  |
| Group x Equation type | 2.44 | .124 | .043 |  | 1.00 | .322 | .018 |
| Group x Difficulty | 0.79 | .378 | .014 |  | 0.05 | .821 | .001 |
| Equation type x Difficulty | 75.7 | < .001 | .579 |  | 48.4 | < .001 | .468 |
| Group x Equation type x Difficulty | 0.08 | .778 | .001 |  | 0.26 | .612 | .005 |
| ***Simple main effects; Equation type x Difficulty*** | | |  |  |  |  |  |
| Multiplication: simple > difficult | 39.2 | < .001 | .416 |  | 187 | <.001 | .773 |
| Subtraction: simple > difficult | 202 | < .001 | .786 |  | 270 | <.001 | .831 |
| Simple: subtraction > multiplication | 4.87 | .032 | .081 |  | .240 | .626 | .004 |
| Difficult: multiplication > subtraction | 66.5 | < .001 | .547 |  | 77.0 | <.001 | .583 |

**Table S2.** Main and interaction effects from the ROI analyses.

|  | lIFG | | |  | lAGp | | |  | | lAGa | | | |  | | | lAG | | |  | | | rHIPS | | |  | | bhippocampus | | |
| --- | --- | --- | --- | --- | --- | --- | --- | --- | --- | --- | --- | --- | --- | --- | --- | --- | --- | --- | --- | --- | --- | --- | --- | --- | --- | --- | --- | --- | --- | --- |
|  | *F* | *p* | *pη^2^* |  | *F* | *p* | *pη^2^* | |  | | *F* | *p* | *pη^2^* | |  | *F* | | *p* | *pη^2^* | |  | *F* | | *p* | *pη^2^* | |  | *F* | *p* | *pη^2^* |
| ***Main effects*** |  |  |  |  |  |  |  | |  | |  |  |  | |  |  | |  |  | |  |  | |  |  | |  |  |  |  |
| Group | 6.79 | 0.012 | 0.11 |  | 3.13 | 0.082 | 0.05 | |  | | 0.92 | 0.342 | 0.02 | |  | 2.58 | | 0.114 | 0.04 | |  | 0.43 | | 0.514 | 0.01 | |  | 0.09 | 0.766 | 0.00 |
| Equation type | 0.02 | 0.877 | 0.00 |  | 12.2 | 0.001 | 0.18 | |  | | 0.08 | 0.785 | 0.00 | |  | 4.50 | | 0.038 | 0.07 | |  | 15.2 | | <.001 | 0.21 | |  | 0.48 | 0.492 | 0.01 |
| Difficulty | 2.54 | 0.117 | 0.04 |  | 2.31 | 0.134 | 0.04 | |  | | 52.6 | <.001 | 0.48 | |  | 21.5 | | <.001 | 0.28 | |  | 17.9 | | <.001 | 0.24 | |  | 1.94 | 0.170 | 0.03 |
| ***Interaction effects*** |  |  |  |  |  |  |  | |  | |  |  |  | |  |  | |  |  | |  |  | |  |  | |  |  |  |  |
| Equation type x Group | 0.33 | 0.567 | 0.01 |  | 2.93 | 0.092 | 0.05 | |  | | 0.13 | 0.716 | 0.00 | |  | 1.32 | | 0.255 | 0.02 | |  | 0.06 | | 0.812 | 0.00 | |  | 2.57 | 0.114 | 0.04 |
| Difficulty x Group | 1.35 | 0.250 | 0.02 |  | 1.74 | 0.193 | 0.03 | |  | | 1.05 | 0.310 | 0.02 | |  | 1.82 | | 0.183 | 0.03 | |  | 0.00 | | 0.970 | 0.00 | |  | 0.71 | 0.403 | 0.01 |
| Equation type x Difficulty | 0.63 | 0.431 | 0.01 |  | 13.1 | 0.001 | 0.19 | |  | | 0.01 | 0.927 | 0.00 | |  | 4.31 | | 0.043 | 0.07 | |  | 0.82 | | 0.370 | 0.01 | |  | 0.05 | 0.830 | 0.00 |
| Equation type x Difficulty x Group | 0.03 | 0.867 | 0.00 |  | 0.22 | 0.643 | 0.00 | |  | | 0.01 | 0.941 | 0.00 | |  | 0.09 | | 0.767 | 0.00 | |  | 1.79 | | 0.186 | 0.03 | |  | 0.53 | 0.469 | 0.01 |

**Table S3.** Resting-state functional connectivity results for all targets. Only connections with FDR-corrected *p* < .001 are shown.

|  | Deaf signers | |  |  |  |  | Hearing non-signers | | |  |  |
| --- | --- | --- | --- | --- | --- | --- | --- | --- | --- | --- | --- |
| Seed-ROI | Target | Conn source | *beta* | *t* | *p_fdr_* |  | Target | Conn source | *beta* | *t* | *p_fdr_* |
| Left inferior frontal gyrus | left IFG (pars opercularis) | atlas | 1.52 | 37.61 | <.001 |  | left IFG (pars opercularis) | atlas | 1.53 | 41.64 | <.001 |
|  | left IFG (pars triangularis) | atlas | 0.97 | 24.05 | <.001 |  | left IFG (pars triangularis) | atlas | 0.99 | 27.75 | <.001 |
|  | left Language IFG | networks | 0.74 | 17.24 | <.001 |  | left Language IFG | networks | 0.76 | 19.05 | <.001 |
|  | left FrontoParietal LPFC | networks | 0.52 | 15.75 | <.001 |  | left frontal operculum | atlas | 0.44 | 14.24 | <.001 |
|  | left cingulo-operculum | atlas | 0.37 | 14.51 | <.001 |  | left MidFG | atlas | 0.41 | 12.82 | <.001 |
|  | left MidFG | atlas | 0.46 | 14.20 | <.001 |  | left Salience AInsula | networks | 0.43 | 12.65 | <.001 |
|  | left toMTG | atlas | 0.40 | 13.08 | <.001 |  | left FrontoParietal LPFC | networks | 0.41 | 11.56 | <.001 |
|  | left pSMG | atlas | 0.46 | 12.80 | <.001 |  | left toMTG | atlas | 0.36 | 11.37 | <.001 |
|  | left planum temporale | atlas | 0.31 | 12.05 | <.001 |  | left Visual Lateral | networks | 0.23 | 10.82 | <.001 |
|  | left Language pSTG | networks | 0.44 | 11.97 | <.001 |  | left iLOC | atlas | 0.23 | 10.37 | <.001 |
|  | left Salience AInsula | networks | 0.47 | 11.89 | <.001 |  | left Language pSTG | networks | 0.36 | 9.94 | <.001 |
|  | left insula | atlas | 0.38 | 11.52 | <.001 |  | right IFG (pars opercularis) | atlas | 0.33 | 9.94 | <.001 |
|  | left pSTG | atlas | 0.41 | 11.30 | <.001 |  | left pSMG | atlas | 0.30 | 8.66 | <.001 |
|  | left Heschl's gyrus | atlas | 0.28 | 10.98 | <.001 |  | right Language IFG | networks | 0.26 | 8.52 | <.001 |
|  | left frontal operculum | atlas | 0.48 | 10.29 | <.001 |  | left toITG | atlas | 0.22 | 8.48 | <.001 |
|  | left parietal operculum | atlas | 0.27 | 10.24 | <.001 |  | left insula | atlas | 0.26 | 8.38 | <.001 |
|  | left PreCG | atlas | 0.37 | 10.23 | <.001 |  | left pSTG | atlas | 0.24 | 8.37 | <.001 |
|  | left aSMG | atlas | 0.40 | 10.22 | <.001 |  | right Language IFG | networks | 0.26 | 8.22 | <.001 |
|  | SensoriMotor Lateral | networks | 0.34 | 9.30 | <.001 |  | left PreCG | atlas | 0.27 | 7.71 | <.001 |
|  | left frontal pole | atlas | 0.28 | 8.78 | <.001 |  | left FOrb | atlas | 0.30 | 7.47 | <.001 |
|  | left Salience SMG | networks | 0.31 | 8.69 | <.001 |  | left sLOC | atlas | 0.21 | 7.44 | <.001 |
|  | left planum polare | atlas | 0.28 | 8.68 | <.001 |  | right iLOC | atlas | 0.15 | 7.35 | <.001 |
|  | left aSTG | atlas | 0.25 | 7.78 | <.001 |  | right Visual Lateral | networks | 0.15 | 6.77 | <.001 |
|  | right IFG (pars opercularis) | atlas | 0.30 | 7.67 | <.001 |  | left TOFusC | atlas | 0.17 | 6.61 | <.001 |
|  | right IFG (pars triangularis) | atlas | 0.23 | 7.41 | <.001 |  | left cingulo-operculum | atlas | 0.25 | 6.53 | <.001 |
|  | right Language IFG | networks | 0.22 | 6.86 | <.001 |  | left aSMG | atlas | 0.25 | 6.49 | <.001 |
|  | left Salience RPFC | networks | 0.20 | 6.63 | <.001 |  | SensoriMotor Lateral | networks | 0.24 | 6.13 | <.001 |
|  | left SFG | atlas | 0.19 | 6.34 | <.001 |  | left Salience SMG | networks | 0.18 | 5.86 | <.001 |
|  | DefaultMode MPFC | networks | -0.20 | -6.23 | <.001 |  | right OFusG | atlas | 0.13 | 5.35 | <.001 |
|  | left angular gyrus | atlas | 0.22 | 6.17 | <.001 |  | left Putamen | atlas | 0.14 | 5.27 | <.001 |
|  | right Hippocampus | atlas | -0.16 | -5.99 | <.001 |  | left DorsalAttention IPS | networks | 0.19 | 5.14 | <.001 |
|  | left FOrb | atlas | 0.25 | 5.99 | <.001 |  | right toITG | atlas | 0.14 | 4.80 | <.001 |
|  | left Pallidum | atlas | 0.15 | 5.95 | <.001 |  | left Heschl's gyrus | atlas | 0.14 | 4.80 | <.001 |
|  | right toMTG | atlas | 0.19 | 5.84 | <.001 |  | left aSTG | atlas | 0.15 | 4.67 | <.001 |
|  | left toITG | atlas | 0.19 | 5.80 | <.001 |  | left Pallidum | atlas | 0.12 | 4.58 | <.001 |
|  | left pMTG | atlas | 0.23 | 5.79 | <.001 |  | DefaultMode MPFC | networks | -0.14 | -4.55 | <.001 |
|  | left Putamen | atlas | 0.17 | 5.75 | <.001 |  | left angular gyrus | atlas | 0.19 | 4.53 | <.001 |
|  | right pSMG | atlas | 0.22 | 5.70 | <.001 |  | left pMTG | atlas | 0.17 | 4.51 | <.001 |
|  | left DorsalAttention IPS | networks | 0.26 | 5.69 | <.001 |  |  |  |  |  |  |
|  | right PostCG | atlas | -0.17 | -5.43 | <.001 |  |  |  |  |  |  |
|  | right FrontoParietal PPC | networks | 0.18 | 5.28 | <.001 |  |  |  |  |  |  |
|  | left FrontoParietal PPC | networks | 0.19 | 5.03 | <.001 |  |  |  |  |  |  |
|  | left iLOC | atlas | 0.17 | 4.96 | <.001 |  |  |  |  |  |  |
|  | left Visual Lateral | networks | 0.15 | 4.76 | <.001 |  |  |  |  |  |  |
|  | right Salience AInsula | networks | 0.16 | 4.74 | <.001 |  |  |  |  |  |  |
|  | right Amygdala | atlas | -0.13 | -4.57 | <.001 |  |  |  |  |  |  |
|  | right Accumbens | atlas | -0.11 | -4.45 | <.001 |  |  |  |  |  |  |
|  | right angular gyrus | atlas | 0.16 | 4.44 | <.001 |  |  |  |  |  |  |
| left anterior angular gyrus | left pSMG | atlas | 0.53 | 14.90 | <.001 |  | left angular gyrus | atlas | 1.33 | 34.45 | <.001 |
|  | left pMTG | atlas | 0.52 | 13.86 | <.001 |  | left FrontoParietal PPC | networks | 1.12 | 28.67 | <.001 |
|  | right FrontoParietal PPC | networks | 0.58 | 13.25 | <.001 |  | left Language pSTG | networks | 0.59 | 21.34 | <.001 |
|  | left Language pSTG | networks | 0.51 | 12.79 | <.001 |  | right FrontoParietal PPC | networks | 0.60 | 19.93 | <.001 |
|  | left sLOC | atlas | 0.57 | 12.43 | <.001 |  | left pMTG | atlas | 0.52 | 18.57 | <.001 |
|  | right angular gyrus | atlas | 0.55 | 11.74 | <.001 |  | right angular gyrus | atlas | 0.62 | 17.79 | <.001 |
|  | left toMTG | atlas | 0.37 | 10.42 | <.001 |  | left sLOC | atlas | 0.56 | 14.70 | <.001 |
|  | left frontal pole | atlas | 0.32 | 10.40 | <.001 |  | left pSMG | atlas | 0.54 | 12.94 | <.001 |
|  | left FrontoParietal LPFC | networks | 0.39 | 8.79 | <.001 |  | left pITG | atlas | 0.31 | 10.09 | <.001 |
|  | right pMTG | atlas | 0.31 | 8.52 | <.001 |  | right DefaultMode LP | networks | 0.38 | 10.00 | <.001 |
|  | right DefaultMode LP | networks | 0.28 | 8.51 | <.001 |  | left DefaultMode LP | networks | 0.36 | 9.32 | <.001 |
|  | right Language pSTG | networks | 0.25 | 8.16 | <.001 |  | right sLOC | atlas | 0.23 | 8.50 | <.001 |
|  | left Language IFG | networks | 0.26 | 7.97 | <.001 |  | right Language pSTG | networks | 0.32 | 8.38 | <.001 |
|  | right pSMG | atlas | 0.27 | 7.84 | <.001 |  | left MidFG | atlas | 0.31 | 8.13 | <.001 |
|  | left SFG | atlas | 0.26 | 7.82 | <.001 |  | right pMTG | atlas | 0.31 | 8.01 | <.001 |
|  | left MidFG | atlas | 0.33 | 7.75 | <.001 |  | left FrontoParietal LPFC | networks | 0.36 | 7.94 | <.001 |
|  | PC | atlas | 0.28 | 7.54 | <.001 |  | right toMTG | atlas | 0.29 | 7.80 | <.001 |
|  | left DefaultMode LP | networks | 0.37 | 7.52 | <.001 |  | left toMTG | atlas | 0.30 | 7.64 | <.001 |
|  | left IFG (pars triangularis) | atlas | 0.19 | 7.08 | <.001 |  | right pSMG | atlas | 0.30 | 7.54 | <.001 |
|  | left pITG | atlas | 0.28 | 6.83 | <.001 |  | left frontal pole | atlas | 0.28 | 7.49 | <.001 |
|  | right toMTG | atlas | 0.23 | 6.78 | <.001 |  | PC | atlas | 0.26 | 7.13 | <.001 |
|  | left aITG | atlas | 0.15 | 6.59 | <.001 |  | right aMTG | atlas | 0.14 | 6.15 | <.001 |
|  | Salience ACC | networks | -0.19 | -5.70 | <.001 |  | left aMTG | atlas | 0.16 | 5.61 | <.001 |
|  | left toITG | atlas | 0.15 | 5.65 | <.001 |  | left Salience SMG | networks | 0.19 | 5.37 | <.001 |
|  | right aMTG | atlas | 0.15 | 5.20 | <.001 |  | left SFG | atlas | 0.18 | 5.29 | <.001 |
|  | right FrontoParietal LPFC | networks | 0.23 | 5.11 | <.001 |  | left Language IFG | networks | 0.19 | 5.18 | <.001 |
|  | right pITG | atlas | 0.18 | 5.03 | <.001 |  | left pSTG | atlas | 0.14 | 5.09 | <.001 |
|  | left FOrb | atlas | 0.15 | 4.85 | <.001 |  | right frontal operculum | atlas | -0.12 | -5.08 | <.001 |
|  | right Cereb2 | atlas | 0.20 | 4.81 | <.001 |  | DefaultMode PCC | networks | 0.19 | 4.99 | <.001 |
|  | right Language IFG | networks | 0.19 | 4.53 | <.001 |  | Precuneous | atlas | 0.20 | 4.99 | <.001 |
|  | right MidFG | atlas | 0.17 | 4.39 | <.001 |  | right pITG | atlas | 0.16 | 4.93 | <.001 |
|  |  |  |  |  |  |  | Salience ACC | networks | -0.17 | -4.73 | <.001 |
|  |  |  |  |  |  |  | left aITG | atlas | 0.13 | 4.62 | <.001 |
| left posterior angular gyrus | left DefaultMode LP | networks | 1.60 | 48.00 | <.001 |  | left DefaultMode LP | networks | 1.66 | 44.75 | <.001 |
|  | left sLOC | atlas | 0.86 | 23.14 | <.001 |  | left sLOC | atlas | 0.87 | 20.99 | <.001 |
|  | right DefaultMode LP | networks | 0.56 | 17.84 | <.001 |  | right DefaultMode LP | networks | 0.67 | 16.88 | <.001 |
|  | left toMTG | atlas | 0.33 | 9.81 | <.001 |  | right sLOC | atlas | 0.38 | 11.86 | <.001 |
|  | left Language pSTG | networks | 0.37 | 9.21 | <.001 |  | left angular gyrus | atlas | 0.42 | 10.58 | <.001 |
|  | DefaultMode PCC | networks | 0.34 | 8.55 | <.001 |  | left Language pSTG | networks | 0.36 | 10.56 | <.001 |
|  | Precuneous | atlas | 0.34 | 8.18 | <.001 |  | DefaultMode PCC | networks | 0.36 | 9.02 | <.001 |
|  | left angular gyrus | atlas | 0.44 | 8.05 | <.001 |  | Precuneous | atlas | 0.36 | 8.84 | <.001 |
|  | PC | atlas | 0.25 | 6.59 | <.001 |  | PC | atlas | 0.25 | 8.06 | <.001 |
|  | right sLOC | atlas | 0.29 | 6.40 | <.001 |  | left toMTG | atlas | 0.31 | 7.97 | <.001 |
|  | left SMA | atlas | -0.12 | -6.39 | <.001 |  | right toMTG | atlas | 0.21 | 7.96 | <.001 |
|  | right toMTG | atlas | 0.19 | 6.23 | <.001 |  | left FrontoParietal PPC | networks | 0.24 | 7.48 | <.001 |
|  | left SCC | atlas | 0.19 | 6.19 | <.001 |  | left pMTG | atlas | 0.26 | 6.99 | <.001 |
|  | left Cuneal | atlas | 0.20 | 6.03 | <.001 |  | left MidFG | atlas | 0.19 | 6.91 | <.001 |
|  | left Visual Lateral | networks | 0.27 | 5.76 | <.001 |  | left Visual Lateral | networks | 0.24 | 6.70 | <.001 |
|  | left aMTG | atlas | 0.18 | 5.50 | <.001 |  | right angular gyrus | atlas | 0.22 | 6.16 | <.001 |
|  | right Language pSTG | networks | 0.17 | 5.25 | <.001 |  | left aMTG | atlas | 0.22 | 6.04 | <.001 |
|  | left FrontoParietal PPC | networks | 0.23 | 4.97 | <.001 |  | left FrontoParietal LPFC | networks | 0.18 | 5.98 | <.001 |
|  | left pSMG | atlas | 0.22 | 4.83 | <.001 |  | right aMTG | atlas | 0.15 | 5.61 | <.001 |
|  | right SMA | atlas | -0.12 | -4.78 | <.001 |  | left pSMG | atlas | 0.20 | 5.54 | <.001 |
|  | left pMTG | atlas | 0.18 | 4.71 | <.001 |  | left pTFusC | atlas | 0.14 | 5.51 | <.001 |
|  | left pPaHC | atlas | 0.12 | 4.65 | <.001 |  | left Cuneal | atlas | 0.18 | 5.34 | <.001 |
|  | left toITG | atlas | 0.16 | 4.52 | <.001 |  | right Visual Lateral | networks | 0.19 | 5.24 | <.001 |
|  | right IFG (pars triangularis) | atlas | 0.13 | 4.49 | <.001 |  | left SCC | atlas | 0.17 | 5.22 | <.001 |
|  |  |  |  |  |  |  | left SMA | atlas | -0.16 | -5.12 | <.001 |
|  |  |  |  |  |  |  | right SMA | atlas | -0.13 | -4.95 | <.001 |
|  |  |  |  |  |  |  | left pPaHC | atlas | 0.14 | 4.89 | <.001 |
|  |  |  |  |  |  |  | left Salience SMG | networks | 0.16 | 4.83 | <.001 |
|  |  |  |  |  |  |  | DefaultMode MPFC | networks | 0.19 | 4.82 | <.001 |
| right horisontal intraparietal sulcus | right FrontoParietal PPC | networks | 0.64 | 21.50 | <.001 |  | right sLOC | atlas | 0.62 | 19.54 | <.001 |
|  | right DorsalAttention IPS | networks | 0.61 | 15.90 | <.001 |  | right DorsalAttention IPS | networks | 0.64 | 18.08 | <.001 |
|  | right angular gyrus | atlas | 0.56 | 15.87 | <.001 |  | right FrontoParietal PPC | networks | 0.68 | 17.80 | <.001 |
|  | right sLOC | atlas | 0.68 | 15.72 | <.001 |  | right pSMG | atlas | 0.57 | 17.07 | <.001 |
|  | right SPL | atlas | 0.62 | 14.28 | <.001 |  | right SPL | atlas | 0.63 | 17.03 | <.001 |
|  | right pSMG | atlas | 0.55 | 13.53 | <.001 |  | right angular gyrus | atlas | 0.57 | 14.48 | <.001 |
|  | right MidFG | atlas | 0.53 | 12.13 | <.001 |  | left FrontoParietal PPC | networks | 0.48 | 13.76 | <.001 |
|  | right toITG | atlas | 0.44 | 12.07 | <.001 |  | left SPL | atlas | 0.44 | 12.43 | <.001 |
|  | left FrontoParietal PPC | networks | 0.49 | 11.15 | <.001 |  | right toITG | atlas | 0.36 | 10.45 | <.001 |
|  | right toMTG | atlas | 0.31 | 10.57 | <.001 |  | right Visual Lateral | networks | 0.33 | 10.37 | <.001 |
|  | left SPL | atlas | 0.41 | 9.63 | <.001 |  | right MidFG | atlas | 0.45 | 10.22 | <.001 |
|  | right FrontoParietal LPFC | networks | 0.50 | 9.09 | <.001 |  | left sLOC | atlas | 0.37 | 9.92 | <.001 |
|  | AC | atlas | -0.27 | -8.97 | <.001 |  | left angular gyrus | atlas | 0.25 | 9.88 | <.001 |
|  | left DorsalAttention IPS | networks | 0.35 | 8.41 | <.001 |  | left pSMG | atlas | 0.29 | 9.82 | <.001 |
|  | right Visual Lateral | networks | 0.32 | 8.39 | <.001 |  | left DorsalAttention IPS | networks | 0.37 | 9.67 | <.001 |
|  | right IFG (pars opercularis) | atlas | 0.25 | 7.86 | <.001 |  | right FrontoParietal LPFC | networks | 0.36 | 8.65 | <.001 |
|  | left sLOC | atlas | 0.39 | 7.78 | <.001 |  | left toITG | atlas | 0.22 | 7.68 | <.001 |
|  | left pSMG | atlas | 0.28 | 7.77 | <.001 |  | right toMTG | atlas | 0.26 | 7.36 | <.001 |
|  | left MidFG | atlas | 0.32 | 7.66 | <.001 |  | right iLOC | atlas | 0.20 | 7.35 | <.001 |
|  | left FrontoParietal LPFC | networks | 0.39 | 7.59 | <.001 |  | AC | atlas | -0.22 | -7.32 | <.001 |
|  | left PaCiG | atlas | -0.17 | -6.87 | <.001 |  | left MidFG | atlas | 0.25 | 7.19 | <.001 |
|  | Cerebellar Anterior | networks | 0.25 | 6.84 | <.001 |  | right aSMG | atlas | 0.26 | 7.08 | <.001 |
|  | left toITG | atlas | 0.29 | 6.77 | <.001 |  | Cerebellar Anterior | networks | 0.20 | 7.06 | <.001 |
|  | DefaultMode MPFC | networks | -0.19 | -6.70 | <.001 |  | left toMTG | atlas | 0.18 | 6.79 | <.001 |
|  | DefaultMode PCC | networks | 0.22 | 6.62 | <.001 |  | left FrontoParietal LPFC | networks | 0.26 | 6.67 | <.001 |
|  | left parietal operculum | atlas | -0.18 | -6.43 | <.001 |  | DefaultMode PCC | networks | 0.21 | 6.53 | <.001 |
|  | right TOFusC | atlas | 0.19 | 6.29 | <.001 |  | left PaCiG | atlas | -0.18 | -6.46 | <.001 |
|  | left angular gyrus | atlas | 0.24 | 5.95 | <.001 |  | left aSMG | atlas | 0.17 | 6.17 | <.001 |
|  | right OFusG | atlas | 0.17 | 5.85 | <.001 |  | right TOFusC | atlas | 0.18 | 6.11 | <.001 |
|  | Cerebellum Ver6 | atlas | 0.13 | 5.81 | <.001 |  | Cerebellum Ver7 | atlas | 0.13 | 6.01 | <.001 |
|  | right DefaultMode LP | networks | 0.25 | 5.66 | <.001 |  | right pITG | atlas | 0.15 | 5.57 | <.001 |
|  | Cerebellum Ver7 | atlas | 0.15 | 5.66 | <.001 |  | left iLOC | atlas | 0.16 | 5.47 | <.001 |
|  | right Cereb6 | atlas | 0.18 | 5.31 | <.001 |  | IFG (pars opercularis) | atlas | 0.20 | 5.47 | <.001 |
|  | right iLOC | atlas | 0.20 | 5.22 | <.001 |  | Precuneous | atlas | 0.17 | 5.41 | <.001 |
|  | right parietal operculum | atlas | -0.15 | -5.21 | <.001 |  | left Visual Lateral | networks | 0.15 | 5.01 | <.001 |
|  | left Amygdala | atlas | -0.11 | -5.17 | <.001 |  | left TOFusC | atlas | 0.13 | 5.00 | <.001 |
|  | left toMTG | atlas | 0.18 | 5.03 | <.001 |  | right DefaultMode LP | networks | 0.20 | 4.96 | <.001 |
|  | right aSMG | atlas | 0.20 | 4.84 | <.001 |  | right Cereb6 | atlas | 0.13 | 4.79 | <.001 |
|  | right SFG | atlas | 0.19 | 4.78 | <.001 |  | left parietal operculum | atlas | -0.12 | -4.62 | <.001 |
|  | right frontal pole | atlas | 0.20 | 4.58 | <.001 |  | left pITG | atlas | 0.12 | 4.60 | <.001 |
|  |  |  |  |  |  |  | left OFusG | atlas | 0.14 | 4.59 | <.001 |
| bilateral hippocampus | left Hippocampus | atlas | 0.99 | 30.05 | <.001 |  | left Hippocampus | atlas | 1.07 | 33.46 | <.001 |
|  | right Hippocampus | atlas | 1.05 | 26.75 | <.001 |  | right Hippocampus | atlas | 1.08 | 27.42 | <.001 |
|  | left aPaHC | atlas | 0.33 | 12.75 | <.001 |  | left pTFusC | atlas | 0.40 | 16.52 | <.001 |
|  | right pPaHC | atlas | 0.34 | 12.48 | <.001 |  | right pTFusC | atlas | 0.41 | 14.44 | <.001 |
|  | right pTFusC | atlas | 0.40 | 12.20 | <.001 |  | left aPaHC | atlas | 0.42 | 11.94 | <.001 |
|  | right aPaHC | atlas | 0.40 | 12.06 | <.001 |  | left pPaHC | atlas | 0.35 | 11.17 | <.001 |
|  | right Amygdala | atlas | 0.38 | 9.78 | <.001 |  | left Amygdala | atlas | 0.43 | 10.79 | <.001 |
|  | left pPaHC | atlas | 0.39 | 9.67 | <.001 |  | right pPaHC | atlas | 0.34 | 10.66 | <.001 |
|  | left pTFusC | atlas | 0.36 | 9.19 | <.001 |  | right aPaHC | atlas | 0.46 | 10.49 | <.001 |
|  | right Salience RPFC | networks | -0.22 | -7.64 | <.001 |  | right Amygdala | atlas | 0.41 | 9.02 | <.001 |
|  | left Amygdala | atlas | 0.35 | 7.55 | <.001 |  | left aMTG | atlas | 0.25 | 7.58 | <.001 |
|  | left Salience RPFC | networks | -0.21 | -7.24 | <.001 |  | left pMTG | atlas | 0.20 | 7.17 | <.001 |
|  | right aMTG | atlas | 0.23 | 6.69 | <.001 |  | right SMA | atlas | -0.16 | -6.81 | <.001 |
|  | right TOFusC | atlas | 0.22 | 6.69 | <.001 |  | left TOFusC | atlas | 0.21 | 6.78 | <.001 |
|  | Salience ACC | networks | -0.19 | -6.35 | <.001 |  | PC | atlas | 0.14 | 6.31 | <.001 |
|  | right aSTG | atlas | 0.16 | 6.17 | <.001 |  | right pITG | atlas | 0.15 | 6.17 | <.001 |
|  | left SMA | atlas | -0.14 | -5.93 | <.001 |  | left SMA | atlas | -0.16 | -6.16 | <.001 |
|  | left aMTG | atlas | 0.24 | 5.67 | <.001 |  | right Salience RPFC | networks | -0.14 | -6.11 | <.001 |
|  | right pITG | atlas | 0.18 | 5.50 | <.001 |  | right TOFusC | atlas | 0.21 | 6.10 | <.001 |
|  | right SFG | atlas | -0.16 | -5.34 | <.001 |  | right aSTG | atlas | 0.18 | 6.00 | <.001 |
|  | left MidFG | atlas | -0.15 | -5.17 | <.001 |  | left LG | atlas | 0.15 | 5.93 | <.001 |
|  | right IFG (pars opercularis) | atlas | -0.14 | -5.12 | <.001 |  | right IFG (pars opercularis) | atlas | -0.12 | -5.83 | <.001 |
|  | DefaultMode MPFC | networks | 0.16 | 5.01 | <.001 |  | right aMTG | atlas | 0.22 | 5.60 | <.001 |
|  | right pMTG | atlas | 0.19 | 4.99 | <.001 |  | left SFG | atlas | -0.17 | -5.46 | <.001 |
|  | right SMA | atlas | -0.16 | -4.90 | <.001 |  | right pMTG | atlas | 0.16 | 5.46 | <.001 |
|  | MedFC | atlas | 0.17 | 4.87 | <.001 |  | left TP | atlas | 0.21 | 5.40 | <.001 |
|  |  |  |  |  |  |  | right SFG | atlas | -0.19 | -5.37 | <.001 |
|  |  |  |  |  |  |  | left pITG | atlas | 0.15 | 5.35 | <.001 |
|  |  |  |  |  |  |  | right Cereb10 | atlas | -0.11 | -5.26 | <.001 |
|  |  |  |  |  |  |  | left aSTG | atlas | 0.20 | 5.24 | <.001 |
|  |  |  |  |  |  |  | MedFC | atlas | 0.13 | 5.23 | <.001 |
|  |  |  |  |  |  |  | left frontal pole | atlas | -0.13 | -5.13 | <.001 |
|  |  |  |  |  |  |  | left planum polare | atlas | 0.17 | 5.12 | <.001 |
|  |  |  |  |  |  |  | right TP | atlas | 0.16 | 4.97 | <.001 |
|  |  |  |  |  |  |  | left Thalamus | atlas | 0.13 | 4.53 | <.001 |
|  |  |  |  |  |  |  | Salience ACC | networks | -0.13 | -4.53 | <.001 |

**Table S4.** Resting-state functional connectivity results for only ROI targets.

|  |  | Deaf signers | |  | Hearing non-signers | |
| --- | --- | --- | --- | --- | --- | --- |
|  |  | *beta* | *p* |  | *beta* | *p* |
| l.IFG | r.HIPS | 0.150 | <.001 |  | 0.096 | 0.011 |
|  | l.pAG | 0.033 | 0.365 |  | 0.094 | 0.005 |
|  | l.aAG | 0.098 | 0.008 |  | 0.076 | 0.052 |
|  | hippocampus | -0.098 | 0.003 |  | -0.067 | 0.009 |
| rHIPS | l.pAG | 0.024 | 0.644 |  | 0.051 | 0.201 |
|  | l.aAG | 0.300 | <.001 |  | 0.290 | <.001 |
|  | hippocampus | 0.002 | 0.944 |  | -0.0001 | 0.996 |
| l.pAG | l.aAG | 0.520 | <.001 |  | 0.510 | <.001 |
|  | hippocampus | 0.070 | 0.089 |  | 0.076 | 0.005 |
| l.aAG | hippocampus | 0.049 | 0.147 |  | 0.034 | 0.202 |

l.IFG left inferior frontal gyrus, l.pAG left posterior angular gyrus, l.aAG

left anterior angular gyrus, rHIPS right horizontal intraparietal sulcus.

**Table S5.** Correlation between performance and mean ROI values in left inferior frontal gyrus.

|  | All participants | | Deaf signers | | Hearing non-signers | |
| --- | --- | --- | --- | --- | --- | --- |
| Task | *r* | *p* | *r* | *p* | *r* | *p* |
| Simple multiplication | .027 | .839 | .156 | .428 | -.349 | .063 |
| Difficult multiplication | .227 | .089 | .120 | .543 | .065 | .737 |
| Simple subtraction | .071 | .598 | .018 | .927 | .027 | .889 |
| Difficult subtration | .130 | .335 | .085 | .668 | -.034 | .859 |


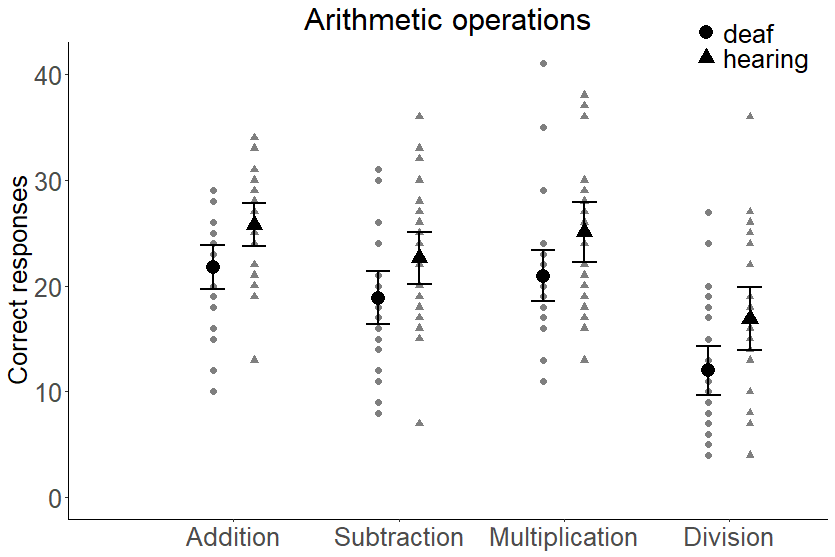


**Figure S1**. Arithmetic skills measured as number of correct answers within 2 minutes for the respective equation type. Error bars represent 95% confidence intercal.


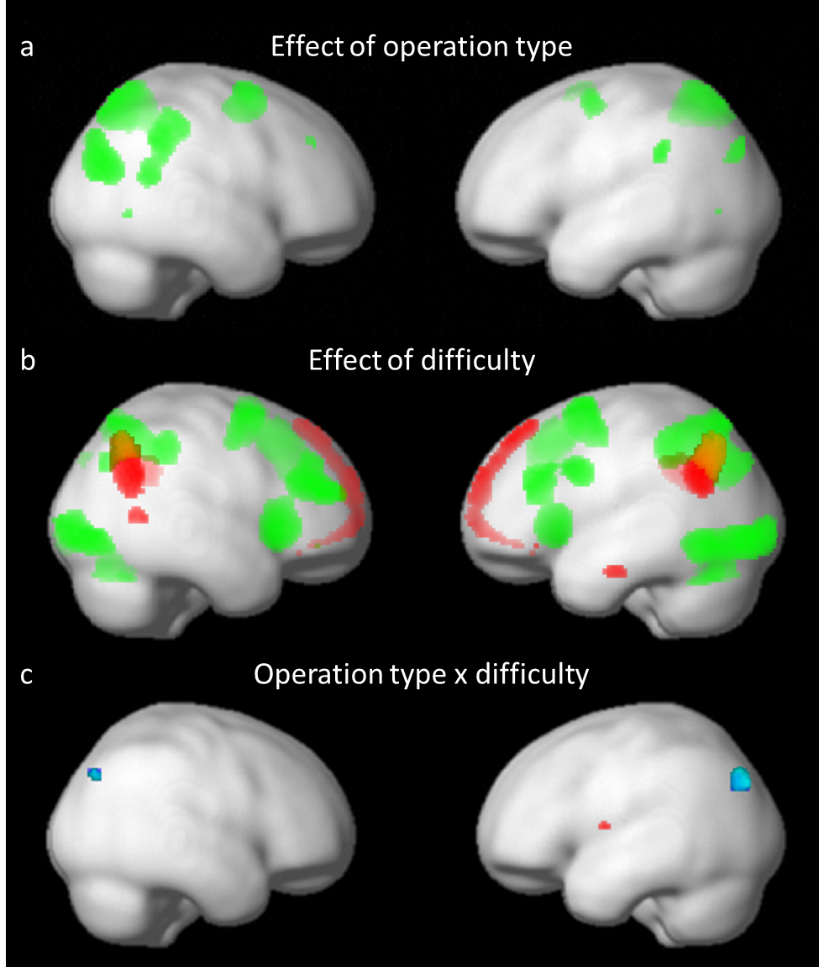


**Figure S2**. A) Effect of equation type; green = subtraction > multiplication within the main effect of equation type contrast, b) effect of difficulty; red = simple > difficult, green = difficult > simple within the main effect of equation type contrast, c) simple main effects; red = subtraction: simple > difficult, green = subtraction difficult > simple, blue = difficulty subtraction > multiplication, turquoise = overlap between green and blue, within the interaction of type and difficulty.
